# Identification and characterization of putative xylose and cellobiose transporters in *Aspergillus nidulans*

**DOI:** 10.1186/s13068-016-0611-1

**Published:** 2016-09-26

**Authors:** Thaila Fernanda dos Reis, Pollyne Borborema Almeida de Lima, Nádia Skorupa Parachin, Fabiana Bombonato Mingossi, Juliana Velasco de Castro Oliveira, Laure Nicolas Annick Ries, Gustavo Henrique Goldman

**Affiliations:** 1 Departamento de Ciências Farmacêuticas, Faculdade de Ciências Farmacêuticas de Ribeirão Preto, Universidade de São Paulo, Av. do Café S/N, Ribeirão Preto, SP CEP 14040-903 Brazil; 2 Departamento de Biologia Celular, Instituto de Biologia, Universidade de Brasília, Brasília, CEP 70910-900 Brazil; 3 Laboratório Nacional de Ciência e Tecnologia do Bioetanol (CTBE), Campinas, Brazil

**Keywords:** *Aspergillus nidulans*, *Saccharomyces cerevisiae*, Xylose, Cellobiose, Sugar transport

## Abstract

**Background:**

The conversion of lignocellulosic biomass to biofuels (second-generation biofuel production) is an environmentally friendlier alternative to petroleum-based energy sources. Enzymatic deconstruction of lignocellulose, catalyzed by filamentous fungi such as *Aspergillus nidulans*, releases a mixture of mono- and polysaccharides, including hexose (glucose) and pentose (xylose) sugars, cellodextrins (cellobiose), and xylooligosaccharides (xylobiose). These sugars can subsequently be fermented by yeast cells to ethanol. One of the major drawbacks in this process lies in the inability of yeast, such as *Saccharomyces cerevisiae*, to successfully internalize sugars other than glucose. The aim of this study was, therefore, to screen the genome of *A. nidulans*, which encodes a multitude of sugar transporters, for transporters able to internalize non-glucose sugars and characterize them when introduced into *S. cerevisiae*.

**Results:**

This work identified two proteins in *A. nidulans*, CltA and CltB, with roles in cellobiose transport and cellulose signaling, respectively. CltA, when introduced into *S. cerevisiae*, conferred growth on low and high concentrations of cellobiose. Deletion of *cltB* resulted in reduced growth and extracellular cellulase activity in *A. nidulans* in the presence of cellobiose. CltB, when introduced into *S. cerevisiae*, was not able to confer growth on cellobiose, suggesting that this protein is a sensor rather than a transporter. However, we have shown that the introduction of additional functional copies of CltB increases the growth in the presence of low concentrations of cellobiose, strongly indicating CltB is able to transport cellobiose. Furthermore, a previously identified glucose transporter, HxtB, was also found to be a major xylose transporter in *A. nidulans*. In *S. cerevisiae*, HxtB conferred growth on xylose which was accompanied by ethanol production.

**Conclusions:**

This work identified a cellobiose transporter, a xylose transporter, and a putative cellulose transceptor in *A. nidulans*. This is the first time that a sensor role for a protein in *A. nidulans* has been proposed. Both transporters are also able to transport glucose, highlighting the preference of *A. nidulans* for this carbon source. This work provides a basis for future studies which aim at characterizing and/or genetically engineering *Aspergillus* spp. transporters, which, in addition to glucose, can also internalize other carbon sources, to improve transport and fermentation of non-glucose sugars in *S. cerevisiae*.

**Electronic supplementary material:**

The online version of this article (doi:10.1186/s13068-016-0611-1) contains supplementary material, which is available to authorized users.

## Background

An increase in energy demands, a depletion in fossil fuels, and high emissions of greenhouse gases have led to the search for alternative and environmentally friendlier energy sources. One alternative energy source is lignocellulose which is found in the cell walls of all plants, such as hardwoods, softwoods, crops, and grasses, thus making it the most abundant organic material on the planet [1–4]. Lignocellulosic wastes are produced by the forestry, pulp and paper, and agriculture industries in addition to municipal and animal wastes [5]. The main components of lignocellulosic biomass are cellulose (40–50 %), hemicelluloses (25–35 %) and lignin (15–20 %) [6]. Cellulose consists of long chains of the hexose sugar glucose, which represents the most abundant simple sugar in the plant cell wall, whereas the main sugar of hemicelluloses is xylose, although other sugars, such as arabinose and galactose, also make up considerable fractions of this polysaccharide. The production of biofuels from lignocellulose, in a process called 2nd generation (2G) biofuel production, aims at converting these sugars into ethanol [7]. Lignocellulosic biomass is deconstructed by enzymatic degradation into a mixture of hexose (e.g., glucose) sugars, pentose (e.g., xylose) sugars, cellodextrins (e.g., cellobiose), and xylooligosaccharides. Cellodextrins are glucose polymers of varying lengths (e.g., cellobiose is a glucose dimer), released during cellulose degradation by cellobiohydrolases and which are subsequently cleaved into glucose monomers by β-glucosidases [5, 8, 9]. Once these simpler sugars have been released from the complex lignocellulosic polymers by enzymatic deconstruction, they can be converted into ethanol by fermenting organisms.

The preferred organism for fermentation of lignocellulosic sugars to ethanol is the budding yeast *Saccharomyces cerevisiae*, which is substantially used in several industrial processes, such as baking, brewing, and wine making [10]. *S. cerevisiae* primarily uses glucose monomers for fermentation and is unable to ferment cellobiose. Furthermore, *S. cerevisiae* is also unable to grow efficiently on xylose as the sole carbon source, although its genome appears to encode all components necessary for metabolizing xylose [11]. Genetic engineering of *S. cerevisiae* has introduced components into the yeast cells that allowed fermentation of cellobiose and xylose [12, 13], but transport of these sugars into the cell is still a limiting factor for successful conversion to ethanol. Complete fermentation of all the sugars found in lignocellulose is desired to reduce the costs of 2G biofuel production and make it an economically feasible process [14]. Therefore, one of the bottlenecks of the conversion of lignocellulose to ethanol lies in the engineering of yeast strains, which can efficiently transport xylose, cellobiose and other lignocellulosic sugars into the cell [15].

Transport of carbon sources is mainly carried out by single polypeptide secondary carriers belonging to the major facilitator superfamily (MFS) of transporters and which transport small soluble molecules in response to ion gradients [16, 17]. The MFS of transporters is divided into 17 families of which families 1, 5, and 7 are involved in sugar transport [16, 17]. Domestic and wild-type *S. cerevisiae* species transport xylose into the cell with low affinity (K_M_ = 100–190 mM) via the expression of native high-affinity hexose transporter-encoding genes, such as *GAL2* and *HXT7* [18, 19], highlighting the preference of *S. cerevisiae* for glucose. Although specific pentose transporters have not been described in yeast, engineering of hexose transporters has been shown to significantly improve xylose transport [20–22]. Furthermore, heterologous introduction of specific d-xylose transporters, derived from other organisms, can improve the growth rate of *S. cerevisiae* on xylose, increasing V_max_ (maximum reaction velocity rate) values and displaying an increase in high cell density sugar consumption [23]. However, this heterologous system only supports low rates of d-xylose transport [24, 25] and may not be perfectly integrated in the endogenous carbon metabolism regulatory network of *S. cerevisiae*. Similar to xylose transporters, cellodextrin transporters from *Neurospora crassa* have also been introduced into *S. cerevisiae* together with a β-glucosidase-encoding gene; they conferred the ability of *S. cerevisiae* to grow on cellobiose [26]. The advantages of *S. cerevisiae* being able to directly use cellobiose for growth are that this does not require adding large quantities of β-glucosidases into the cultures and it also prevents the build-up of glucose in the culture medium that is repressive for cellulase and hemicellulase-encoding genes [27]. However, further engineering is required to optimize cellobiose transport and metabolism in *S. cerevisiae*. The search to find xylose and cellobiose-specific transporters is, therefore, of importance for bioethanol production from lignocellulose.

Filamentous fungi degrade lignocellulosic biomass through secreting a large repertoire of hydrolytic enzymes that break down lignocellulosic sugar polymers into simple sugars which subsequently can be taken up by the cell [28, 29]. Accordingly, the genomes of filamentous fungi also encode large numbers of MFS transporters. Currently, the genomes of *Trichoderma reesei* and *A. nidulans* are predicted to encode 164 and 357 proteins, respectively, belonging to the MFS, although it is not known how many of these are exactly involved in sugar transport [30, 31]. In addition, filamentous fungi, such as *N. crassa* and *T. reesei*, are able to transport disaccharides such as cellobiose into the cell through cellobiose-specific transporters; once internalized, cellobiose has been shown to play an important role in signaling the presence of cellulose [12, 19, 32]. Furthermore, transporters expressed by filamentous fungi often can transport more than one type of sugar; for example, the *A. nidulans* transporter XtrD was shown to be able to transport, in addition to xylose and glucose, several other monosaccharides, whereas the *T. reesei* STP1 transporter is involved in glucose and cellobiose uptake [32, 33]. However, a very few sugar transporters have been functionally characterized in filamentous fungi [34–40].

The aim of this work was, therefore, to identify and characterize *A. nidulans* transporters involved in cellobiose and xylose uptake and heterologously introduce them into *S. cerevisiae*. This study identified several transporters with roles in pentose or cellodextrin transport. Characterization of the cellobiose transporter CltA showed increased efficiency in cellobiose transport than when compared to a previously identified *N. crassa* cellobiose transporter. Furthermore, this work identified CltB as a putative cellobiose transceptor. In addition, a previously described glucose transporter was identified as playing a major role in xylose transport.

## Results

### Identification of CltA and CltB with roles in cellobiose transport or signaling

We have previously used genome-wide transcriptional profiling to identify 12 transporters, belonging to the major facilitator superfamily (MFS) that have increased mRNA accumulation in xylose-rich conditions [33]. One of these transporters, named XtrD, was identified as a xylose-specific transporter [33]. We, therefore, started by characterizing three other randomly chosen transporters (*xtrF*-*H* that correspond to AN0332, AN8347, and AN9173, respectively) that belong to this series of putative xylose transporters. Although these three genes were upregulated in the presence of xylose, deletion of these genes in *A. nidulans* did not have a significant effect on growth in the presence of xylose and glucose nor could they confer growth, when heterologously introduced, of *S. cerevisiae* in the presence of xylose, glucose, and other monosaccharides (data not shown). Since these transporters were not able to transport either hexoses or pentoses, we hypothesized if they could be involved in the transport of more complex sugars, such as cellodextrins (e.g., cellobiose) or xylooligosaccharides. The genome of *N. crassa* encodes two cellobiose transporters termed *CDT*-*1* and *CDT*-*2* which transport and internalize cellodextrin molecules [26, 41] and which also appear to have transceptor activity and, therefore, play a role in cellulose signaling [42]. The here identified, supposed xylose transporter-encoding gene *xtrG* (AN8347) has identity with the *N. crassa* cellobiose transporter *CDT*-*2* (44 % identity, 61 % similarity, e-value of 4e−137). BLASTp search of the *Aspergillus* genome database (www.aspgd.org) using *N. crassa CDT*-*1* as a query allowed us to identify a second gene, AN2814, with high identity to the *N. crassa* cellodextrin transporter (61 % identity, 75 % similarity, e-value of 0.0).

**Fig. 1:**
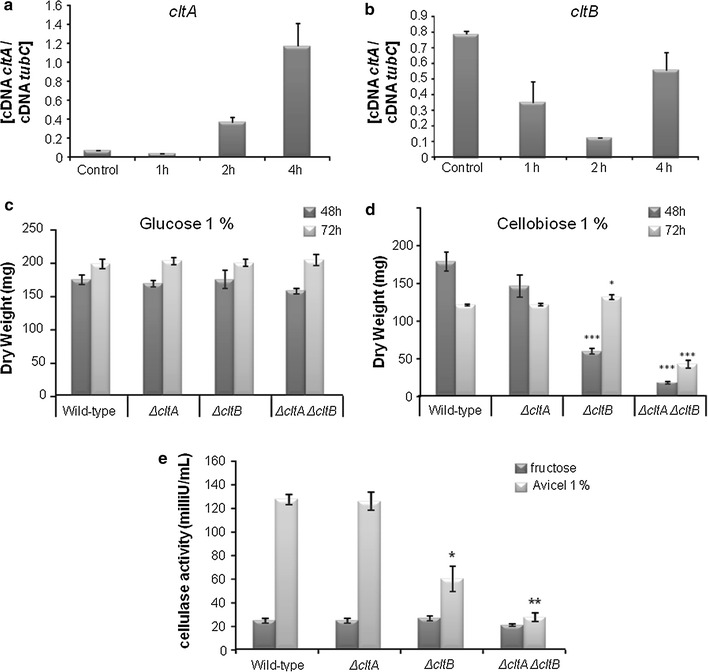
CltA and CltB are cellobiose transporters which are involved in the signaling response to cellulose. The expression of *cltA* (**a**) and *cltB* (**b**) was assessed by RT-qPCR in the presence of 1 % cellobiose. The effect of the single deletions of *cltA* and *cltB* and the double deletion *cltA cltB* was assessed on fungal biomass accumulation (*DW* dry weight) when grown for 48 or 72 h in the presence of 1 % glucose (**c**) or 1 % cellobiose (**d**). Cellulase activity (**e**) was measure in the supernatants of the same strains when grown in minimal medium supplemented with 1 % fructose for 16 h and then transferred to minimal medium containing 1 % avicel for 5 days. *Error bars* indicate standard deviation for three biological replicates

To further characterize these potential cellobiose transporter-encoding genes (here now named *cltA* and *cltB*, respectively), we evaluated their expression patterns in the presence of 1 % cellobiose (Fig. 1a, b). The expression of *cltA* increased gradually (about 4.8-fold) over a time period of 4 h, whereas expression of *cltB* varied during the same time period (Fig. 1a, b). Next, both genes were deleted in *A. nidulans* and a Δ*cltA* Δ*cltB* double deletion strain was constructed. The wild-type, *ΔcltA*, *ΔcltB,* and the double *ΔcltA* Δ*cltB* strains were grown in 1 % glucose and 1 % cellobiose for 48 and 72 h, and biomass was determined (Fig. 1c, d). All the mutant strains had a similar biomass than the wild-type strain when grown in 1 % glucose (Fig. 1c). However, in the presence of cellobiose, the *ΔcltB* strain showed a ~50 % reduction in biomass after 48 h growth when compared to the wild-type strain, whereas there was no significant difference between the Δ*cltA* and wild-type strains (Fig. 1d). The double mutant showed a ~75 % reduction in biomass when compared to the wild-type strain after 48-h growth in 1 % cellobiose (Fig. 1d). These results suggest that CltA and CltB could collaborate towards cellobiose transport. Interestingly, there is also a reduction in cellulase activity in the *ΔcltB* and *ΔcltA* Δ*cltB* mutants of 50 and 70 %, respectively, than when compared to the wild-type strain (Fig. 1e), suggesting that these transporters play a role in the regulation/signaling of cellulase production.

**Fig. 2:**
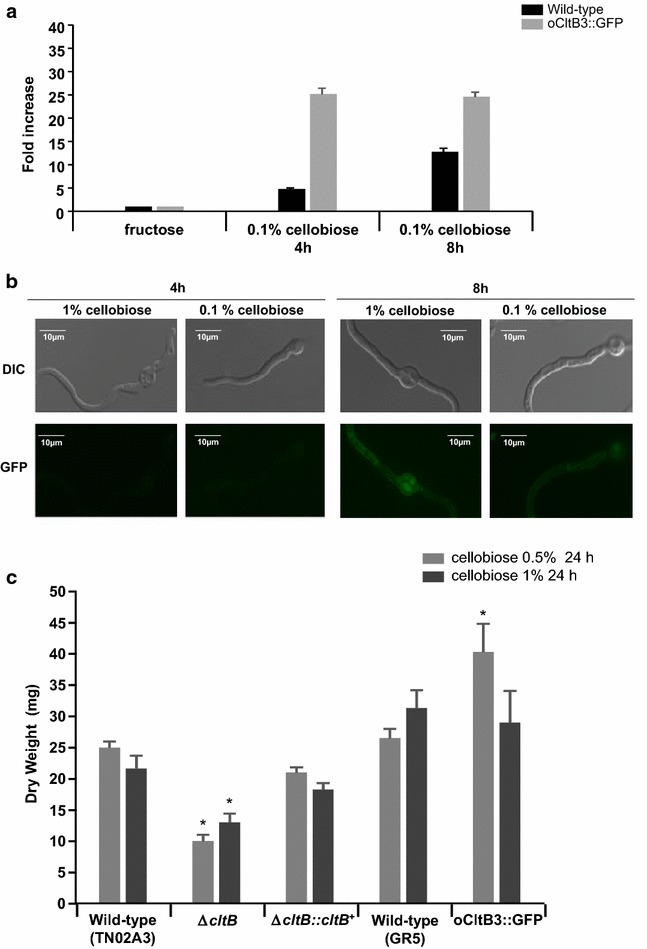
CltB overexpression increases the growth on cellobiose. **a** The expression of *cltB* was assessed by qRT-PCR in the presence of 0.1 % cellobiose. **b** Germlings of oCltB3::GFP were grown for 16 h in fructose 1 % and transferred to 0.1 or 1 % cellobiose. **c** The fungal biomass accumulation (dry weight) in the wild-type (TN02A3 and GR5), Δ*cltB*, Δ*cltB::cltB* ^+^, and oCltB3::GFP strains was assessed for 24 h in the presence of 0.5 or 1 % cellobiose. *p < 0.005

We decided to investigate in more detail the phenotype provided by Δ*cltB* by complementing and overexpressing the *cltB*. First, we complemented the Δ*cltB* with a wild-type copy of *cltB* integrated ectopically, creating a strain Δ*cltB::cltB*^+^. Subsequently, we transformed the wild-type GR5 strain with CltB::GFP and selected for transformants with a single homologous integration and multiple ectopic integrations (Additional file 1). We selected single candidates for homologous (named CltB::GFP) and multiple ectopic integrations (named oCltB3::GFP). Growth phenotypes of Δ*cltB::cltB*^+^, CltB::GFP, and oCltB3::GFP were identical to the wild-type strain on MM with glucose as single carbon source (data not shown). Expression measured by qRT-PCR experiments showed that oCltB3::GFP has about eightfold more *cltB* expression than the wild-type strain in the presence of cellobiose (Fig. 2a). To verify the cellular localization and expression of CltB:GFP, the GFP strain was grown for 16 h in fructose and transferred to either 0.1 or 1 % cellobiose for 4 or 8 h (Fig. 2b). We have not observed any fluorescence in fructose (data not shown), but in contrast in 1 % cellobiose, we were able to see a weak fluorescence in oCltB3::GFP, mostly localized in the cytoplasm and in the cell membrane (Fig. 2b). To evaluate the impact of overexpressing *cltB*^+^ on growth in the presence of 0.5 and 1 % cellobiose as a single carbon source, the wild-type, Δ*cltB*, Δ*cltB::cltB*^+^, and oCltB3::GFP were grown for 24 h in MM + 0.5 or 1 % cellobiose (Fig. 2c). There is no significant difference in the growth (as evalutated by dry weight) of the wild type and Δ*cltB::cltB*^+^ in both 0.5 and 1 % cellobiose (Fig. 2c); in contrast, as it is also shown in Fig. 1d, we have observed a significant differential reduced growth in Δ*cltB* in both cellobiose concentrations (Fig. 2c). The overexpression strain oCltB3::GFP has shown more growth than the wild type only in 0.5 % but not in 1 % cellobiose (Fig. 2c). Taken together, these results suggest that CltB is able to transport cellobiose.

### CltA and CltB confer the ability of *S. cerevisiae* to grow in the presence of cellobiose as sole carbon source

**Fig. 3:**
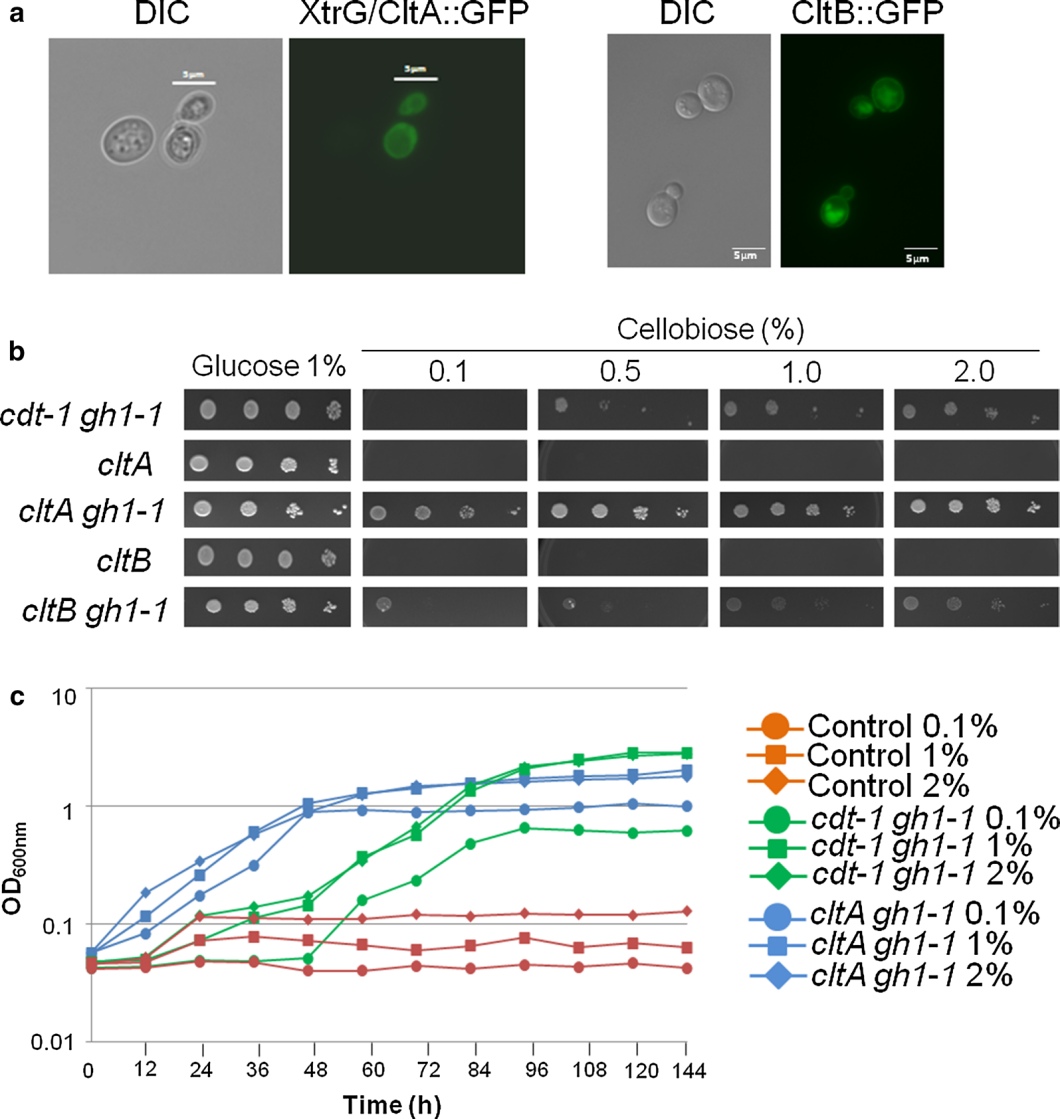
CltA and CltB confer growth of *S. cerevisiae* in the presence of low and high concentrations of cellobiose. **a** CltA and CltB localize to the plasma membrane in *S. cerevisiae*. Strains were grown for 16 h at 30 °C in YNB supplemented with 2 % glucose before pictures were taken without (DIC) and with (GFP) fluorescence. **b** *S. cerevisiae* strains containing *A. nidulans cltA*, *cltB,* and *N. crassa cdt*-*1* with and without the β-glucosidase-encoding (*gh1*-*1*) gene were pre-grown for 24 h in YNB media containing 1 % glucose before a serial dilution was made (1:10 dilution starting at optical density OD_600nm_ 1.0) and diluted cells were grown on YNB plates containing different concentrations of cellobiose (0.1–2 %). **c** Growth curves of *S. cerevisiae* strains containing the *N. crassa cdt*-*1* and the *A. nidulans cltA* transporter-encoding genes. Both strains also contained the β-glucosidase-encoding gene *gh1*-*1*. Strains were grown for 144 h in the presence of different concentrations of cellobiose (0.1, 1, and 2 %) at 30 °C. Growth was assessed by measuring the OD at 600 nm. The yeast strain expressing CltB has not grown in liquid medium, and it was not used in this experiment

To evaluate the ability of CltA and CltB to transport cellobiose, both genes were cloned into *S. cerevisiae* SC9721_pGH1-1, a SC9721 strain previously transformed with the *N. crassa* β-glucosidase-encoding gene *gh1*-*1* (NCU00130) [26]. Both *cltA* and *cltB* were fused to *gfp,* and plasma membrane localization of CltA and CltB was confirmed by fluorescence microscopy when grown for 24 h in YNB supplemented with 1 % glucose medium (Fig. 3a). The *S. cerevisiae* CltA::GFP and CltB::GFP strains were then grown in liquid YNB medium supplemented with 1 % glucose for 24 h at 30 °C, before cells were washed and spotted in a serial dilution onto YNB solid medium containing either 1 % glucose or varying concentrations of cellobiose. Yeast strains containing only CltA or CltB (no β-glucosidase) were used as negative controls as they were unable to grow on cellobiose as sole carbon source. *S. cerevisiae* transformed with *N. crassa cdt*-*1* and the β-glucosidase-encoding gene (*gh1*-*1*) was used as a positive control (Fig. 3b). The drop-out assay clearly shows that CltA, and to a lesser extent CltB, is able to transport cellobiose and, thus, enable *S. cerevisiae* to grow on cellobiose as sole carbon source.

To compare the efficiency of these cellobiose transporters, the growth rates of *S. cerevisiae* strains harboring either the *A. nidulans* CltA or the *N. crassa* CDT-1 transporters were compared in the presence of different concentrations of cellobiose. Although *cltA* was expressed from a weaker yeast promoter than *cdt*-*1* [43–45], the *S. cerevisiae* CltA strain grew much faster during the first 36 h incubation in different concentrations of cellobiose, suggesting that CltA appears to transport different concentrations of cellobiose faster into the yeast cell than when compared to the yeast strain containing CDT-1 (Fig. 3c).

### Deletion of the hexose transporter-encoding gene hxtB results in reduced xylose uptake

As mentioned in the introduction, many sugar transporters expressed by filamentous fungi are capable of transporting more than one type of monosaccharide across the fungal membrane. As a next step, we decided to investigate the possibility that *A. nidulans* hexose transporters could be involved in xylose uptake. In a previous study, four hexose transporters, termed HxtB-E were shown to confer growth of *S. cerevisiae* strain EBY.VW4000 in the presence of glucose, fructose, mannose, and galactose [46]. These transporters, therefore, seem to accept multiple sugars as substrates, although xylose as a potential substrate for these transporters was not characterized at that time. In *A. niger,* on the other hand, MstA, the orthologue of HxtB in *A. nidulans* was shown to have high affinity for xylose when introduced into *S. cerevisiae* [47]. We, therefore, decided to investigate whether these four transporters were able to transport xylose into the cell.

**Fig. 4:**
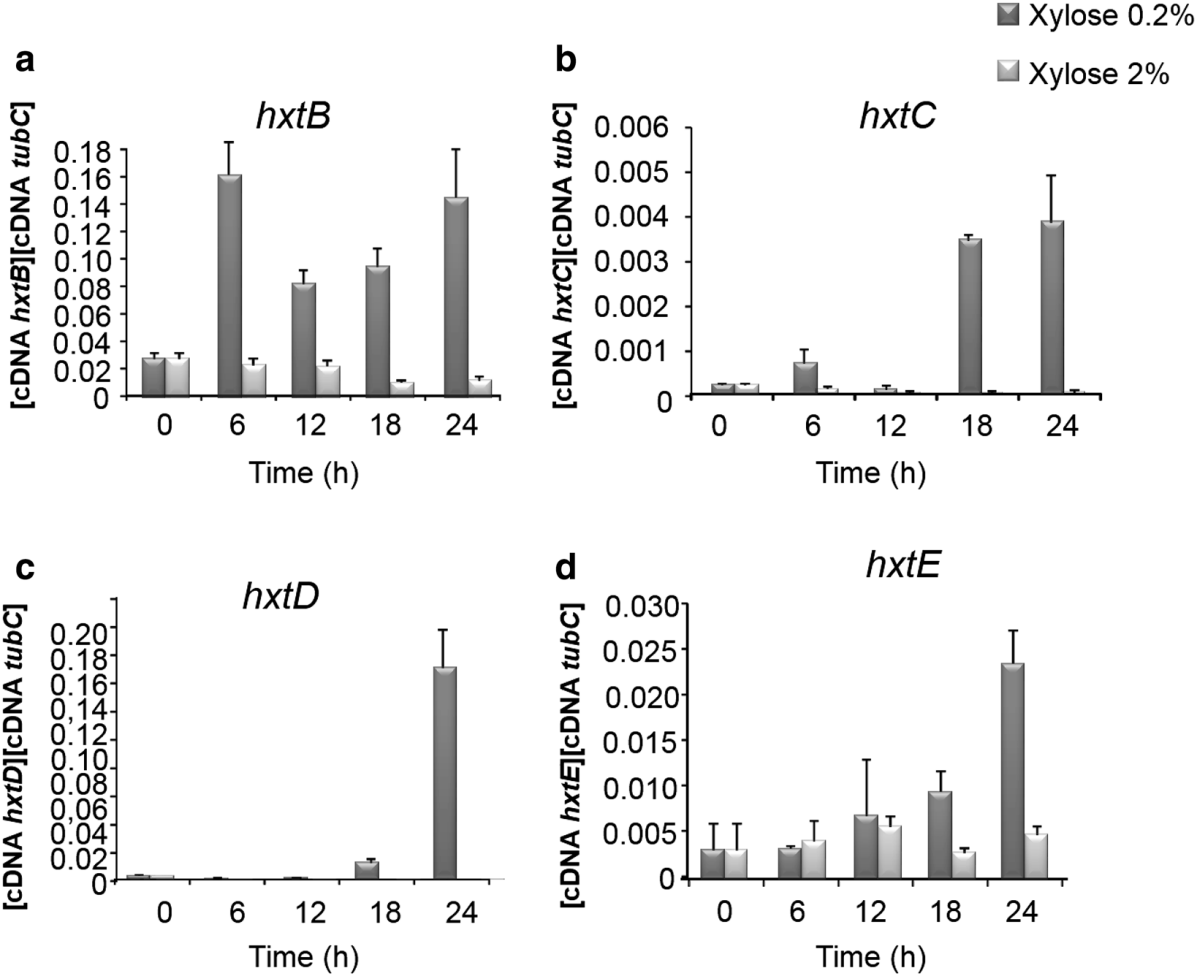
The expression of the *A. nidulans* hexose transporter-encoding genes *hxtB*-*E* is upregulated in the presence of low concentrations of xylose. Transcript levels of *hxtB*-*E* (**a**–**d**) were determined by RT-qPCR in the wild-type strain when grown for 0, 6, 12, 18, and 24 h in the presence of 0.2 and 2 % xylose*. Error bars* indicate the standard deviation for three replicates

**Fig. 5:**
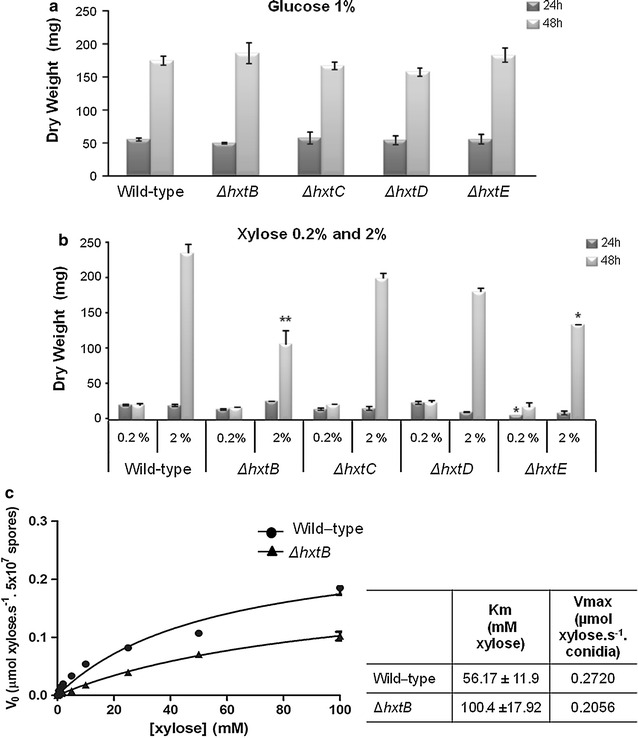
HxtB is a xylose transporter. Fungal dry weight of the wild-type, Δ*hxtB*, Δ*hxtC*, Δ*hxtD,* and Δ*hxtE* strains was measured after strains were grown for 24 or 48 h in the presence of 1 % glucose (**a**) or in the presence of 0.2 and 2 % xylose (**b**). Xylose transport, as assessed by ^14^C-xylose uptake, was measured in the wild-type and Δ*hxtB* strains in the presence of different concentrations of xylose (**c**)

The *A. nidulans* wild-type strain was first grown from spores in fructose-rich media before being transferred to media containing either 0.2 or 2 % xylose for 6, 12, 18 and 24 h (Fig. 4). Gene expression of *hxtB*-*E* was assessed by RT-qPCR in these conditions. All four genes were induced to a different extent in the presence of low concentrations of xylose (0.2 %) but not in the presence of 2 % xylose (Fig. 4a–d). Next, the four transporter-encoding genes were knocked out in *A. nidulans* and growth of these strains in the presence of glucose and xylose was assessed. The wild-type and the four deletion strains were grown in liquid minimal medium supplemented with 1 % glucose, 0.2 % xylose or 2 % xylose for 24 and 48 h before fungal dry weight was measured (Fig. 5). All strains showed a similar biomass when grown in 1 % glucose for 24 and 48 h (Fig. 5a). The Δ*hxtB* and Δ*hxtE* strains showed significantly reduced biomass when grown in 2 % xylose for 48 h (Fig. 5b). However, after 72 h of growth, all the mutant strains had a similar dry weight to the wild-type strain (data not shown). To further characterize xylose uptake, the concentration of xylose was measured in the supernatants of the wild-type, Δ*hxtB* and Δ*hxtE* strains when grown for 72 h in medium supplemented with either 1 or 2 % xylose. After 72 h, the wild-type strain and Δ*hxtE* strains had consumed all the xylose in the extracellular medium, whereas xylose consumption was much slower in the Δ*hxtB* strain and residual xylose could still be detected after 72 h in the supernatant of this strain (Table 1).

**Table 1:** Residual xylose in the supernatant during *A. nidulans* growth

Time (h)	Wild-type	*ΔhxtB*	*ΔhxtE*
1 % xylose			
0	100	100	100
24	58.7 ± 6.6	60.7 ± 2.1	56.5 ± 2.0
48	23.8 ± 1.6	49.9 ± 7.5*	27.8 ± 0.2
72	0	17.4 ± 2.9*	0
2 % xylose			
0	100	100	100
24	75.9 ± 3.7	85.6 ± 4.2	73.3 ± 1.6
48	15.4 ± 4.6	37.3 ± 0.6*	19.3 ± 1.2
72	0	6.2 ± 3.4*	0

* *p* < 0.01

To confirm the above described results, the capacity of xylose uptake was studied in both the wild-type and Δ*hxtB* strains using ^14^C-xylose. In the wild-type strain, ^14^C-xylose uptake obeyed single saturation kinetics with a *K*_*m*_ value of 56.17 ± 11.9 mM and a *V*_max_ of 0.27 µmol of xylose h^−1^ per 2.5 × 10^7^ conidia (Fig. 5c; Additional file 2 for Eadie-Hofstee and Lineweaver–Burk plots). The *ΔhxtB* mutant strain showed both a decreased affinity for xylose (*K*_*m*_ = 100.4 ± 17.92 mM) and a slight reduction in transport capacity (*V*_max_ = 0.20 µmol of xylose per hour per 2.5 × 10^7^ conidia; Fig. 5c; Additional file 1 for Eadie-Hofstee and Lineweaver–Burk plots).

### HxtB confers growth of *S. cerevisiae* in the presence of xylose

**Fig. 6:**
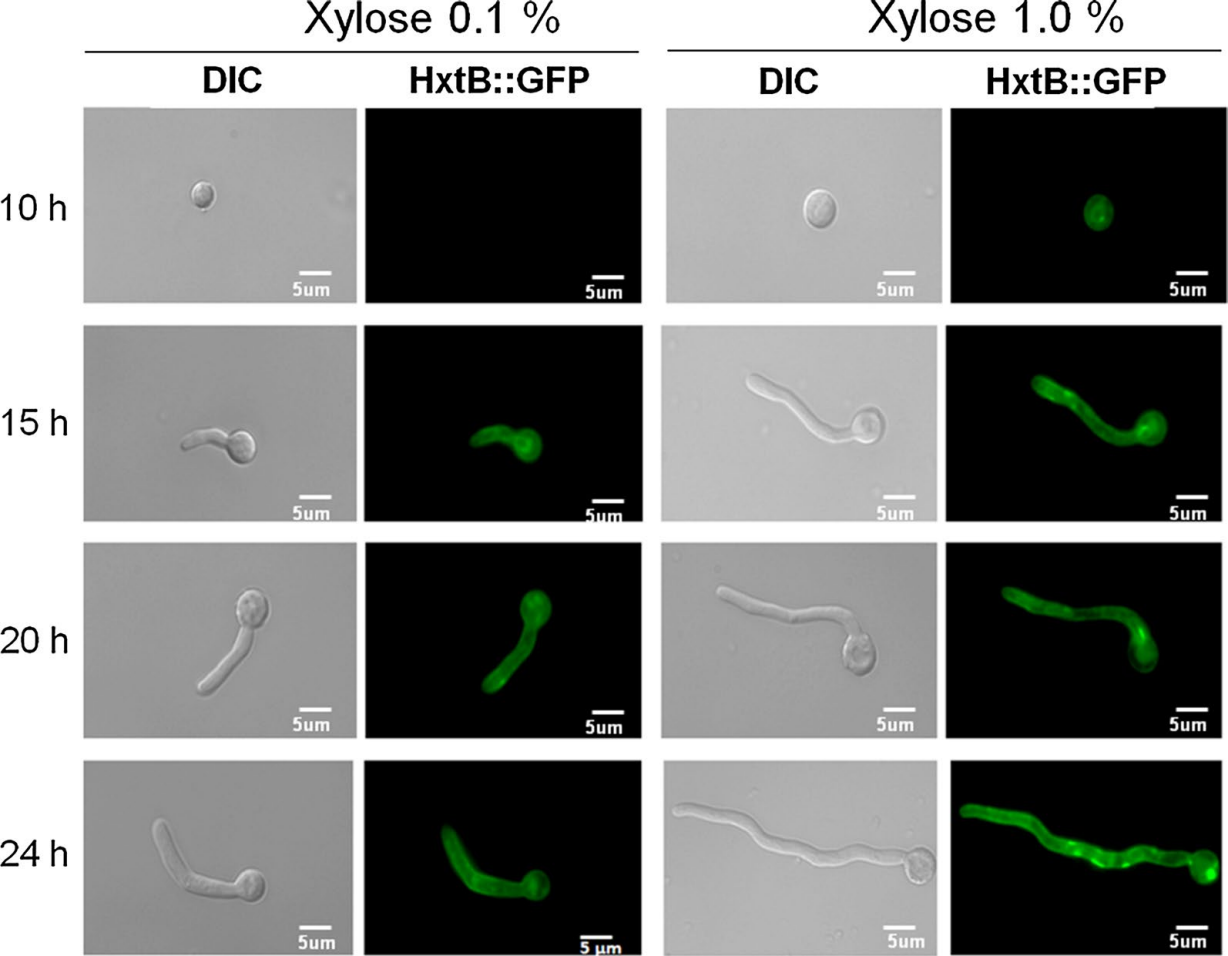
HxtB localizes to the plasma membrane in *A. nidulans* in the presence of xylose. The *A. nidulans* HxtB::GFP strain was grown from conidia in minimal media supplemented with 0.1 or 1 % of xylose for 10, 15, 20, and 24 h. DIC (differential interference contrast) was applied to view unstained hyphae

**Fig. 7:**
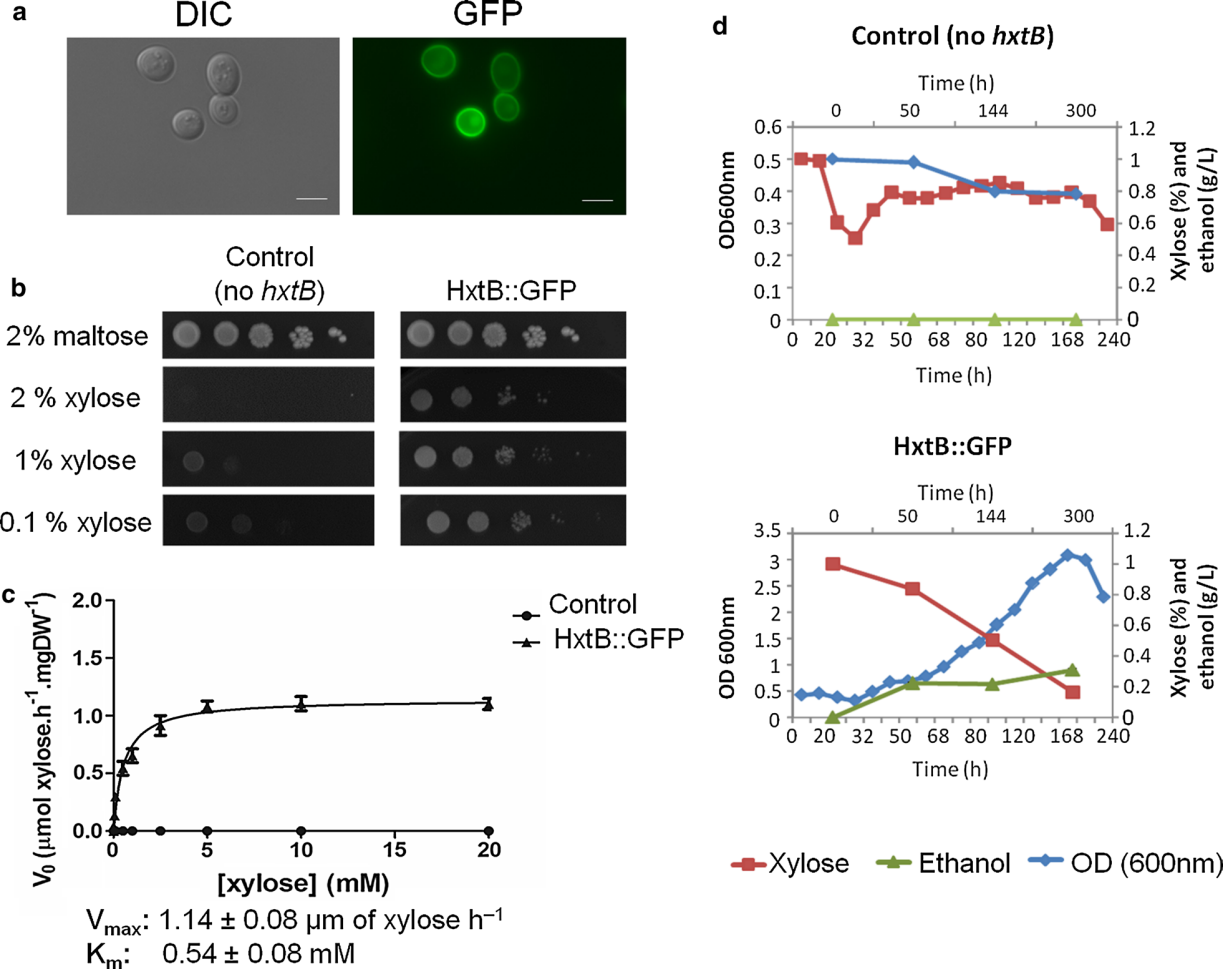
HxtB confers growth of *S. cerevisiae* in the presence of xylose. **a** HxtB::GFP localizes to the plasma membrane in *S. cerevisiae* when grown in maltose-rich media. Microscopy pictures were taken in the absence (DIC) and presence of fluorescence (GFP). **b** Growth of *S. cerevisiae* strain EBY.VW4000 transformed with (*hxtB*) and without (control) the hexose transporter *hxtB* gene in the presence of low (0.1 %) and high (1 %) concentrations of xylose. Both the control and *hxtB* strains also contained the genes encoding enzymes of the xylose metabolic pathway. Yeast strains were first pre-grown in maltose-rich media before a serial dilution was made and cells were incubated on plates containing the different carbon sources at 30 °C for 144 h. **c** Xylose transport as determined by [^14^C] xylose uptake in the *S. cerevisiae hxtB* strain in the presence of different concentrations of xylose. A *S. cerevisiae* strain which did not contain the HxtB hexose transporter was used as the control strain. Standard deviations were obtained from three biological replicates. **d** Growth, as determined by optical density at 600 nm (OD_600nm_), ethanol production and xylose consumption of the *S. cerevisiae* control (does not contain *hxtB*) and HxtB strains when incubated in YNB supplemented with 1 % xylose

To confirm the presence and the cellular localization of HxtB, the HxtB::GFP and HxtE::GFP strains were constructed. Growth phenotypes of HxtB::GFP and HxtE::GFP were identical to the wild-type strain (data not shown). Both strains were grown for 10, 15, 20, and 24 h in minimal medium containing 0.1 or 1 % xylose. HxtB::GFP and HxtE::GFP were expressed in the presence of low and high concentrations of xylose upon which it localized to the fungal plasma membrane and small vacuoles (Fig. 6; Additional file 3). To confirm the xylose-transporting capacity of HxtB::GFP, it was introduced into *S. cerevisiae* EBY.VW4000 strain which was previously transformed with all the components necessary for the xylose metabolic pathway (see “Methods” section). The *S. cerevisiae* EBY.VW4000 strain lacks around 20 glucose transporters and is unable to grow on various hexose and pentose monosaccharides, including glucose, fructose, mannose, galactose, and xylose [48]. This strain is, therefore, a good tool for evaluating the ability of heterologous introduced transporter to take up various sugars thus conferring growth to *S. cerevisiae* in the presence of various pentose and hexose sugars. HxtB::GFP localized to the plasma membrane in *S. cerevisiae* when grown in maltose-rich conditions (Fig. 7a). Furthermore, when transferred from maltose-rich media to media containing low and high concentrations of xylose, *S. cerevisiae* strain HxtB::GFP was able to grow in both 0.1 and 1 % xylose, whereas the strain which lacked HxtB::GFP (control) was not able to do it (Fig. 7b). Furthermore, ^14^C-xylose uptake in *S. cerevisiae* HxtB::GFP followed single saturation kinetics with a *K*_*m*_ value of 0.54 ± 0.08 mM and a *V*_max_ of 1.14 ± 0.08 µm of xylose h^−1^ per mg cell dry weight (Fig. 7c). In contrast, the *S. cerevisiae* strain which does not contain *hxtB* was unable to transport xylose (Fig. 7c). In agreement, *S. cerevisiae* HxtB::GFP was able to grow and consume around 90 % of extracellular xylose after 192 h of growth in xylose-rich medium and at the same time produce ethanol (Fig. 7d). The control strain, which did not contain the HxtB transporter, did not grow in the presence of xylose and, hence, did not consume xylose and did not produce ethanol (Fig. 7d). In addition, as previously reported, *S. cerevisiae* strains containing the transporters HxtB, HxtC, and HxtE are able to grow in the presence of glucose, galactose, fructose, and mannose, while HxtD was not able to use any of these monosaccharides for growth [46]. In contrast, we were not able to see any xylose transport by *S. cerevisiae* strains harboring HxtC, -D, or -E (data not shown). Taken together, these results suggest that HxtB plays, in addition to being a glucose transporter, a major role in xylose uptake.

## Discussion

One of the major drawbacks in biofuel production from lignocellulosic plant material is the inability of fermenting organisms to produce ethanol when growing on sugars other than glucose. Lignocellulose is composed of hexose (glucose) and pentose sugars (mainly xylose) and enzymatic deconstruction of it by, for example, filamentous fungi results in the release of these monosaccharides as well as in the release of oligosaccharides (e.g., the glucose dimer cellobiose) [6]. More specifically, most fermenting organisms are not very efficient at transporting pentose sugars and oligosaccharides into the cell. Complete conversion of all the sugars found in lignocellulose is desired to make 2G biofuel production an economically feasible process [14]. *S. cerevisiae* is one of the preferred organisms for fermentation as it is already applied in various industrial processes and is generally regarded as safe [10].

One successful strategy to improve non-glucose uptake in *S. cerevisiae* is to introduce transporters, from other organisms into its genome. The genomes of filamentous fungi, which are able to internalize a wide variety of mono- and oligosaccharides, are, therefore, screened to find transporters which are able to transport non-glucose sugars. Although this has greatly improved the ability of *S. cerevisiae* to take up pentose sugars such as xylose or cellodextrins such as cellobiose [13, 20, 21, 26], further genetic engineering is required to optimize non-glucose sugar transport. In addition, most transporters encoded by filamentous fungi have not been characterized yet, although these organisms also play a major role in 2G biofuel production. This work, therefore, aimed at identifying xylose- and cellobiose-specific transporters through screening the genome of the filamentous fungus *A. nidulans* and characterizing them when introduced into *S. cerevisiae*.

A BLAST analysis of XtrG, encoded by *xtrG* and identified as being upregulated in the presence of xylose [33], showed similarity to the N. crassa cellobiose transporter CDT-2. The name of XtrG was subsequently changed to CltA (cellobiose transporter A). Furthermore, another *A. nidulans* protein, encoded by AN2814, showed high identity to the *N. crassa* cellodextrin transporter CDT-1 and was, therefore, termed CltB. Cellooligosaccharides, such as cellobiose, released during enzymatic degradation of cellulose, have been shown to be important molecules for cellulase gene induction in filamentous fungi, such as *T. reesei, N. crassa,* and *P. oxalicum* [49–53]. The expression of *cltA* increased in the presence of cellobiose. Deletion of *cltB* and of *cltA* and *cltB* simultaneously, but not of *cltA*, resulted in reduced growth and cellulase secretion in the presence of cellobiose during the first 48 h, although this growth was restored after 72 h in the Δ*cltB* strain. In contrast, when introduced into *S. cerevisiae* strain SC9721 together with a β-glucosidase-encoding gene, CltB conferred only slow growth in the presence of different concentrations of cellobiose. These results suggest that the main function of CltB may not be cellobiose transport; it may also function as a transceptor involved in signaling the presence of lignocellulosic biomass. However, we have shown that the introduction of additional functional copies of CltB increases the growth in the presence of low concentrations of cellobiose, strongly indicating CltB is able to transport cellobiose. In *N. crassa*, CDT-1 and CDT-2, in addition to being cellobiose transporters, have been hypothetized to having a role in downstream signaling upon the detection of cellulose by the fungus [42]. Furthermore, the *T. reesei* transporters STP1 and CRT1 were also proposed to play an important role in the induction of cellulase-encoding genes [32]. This is the first time that a potential transceptor role has been identified for a protein in *A. nidulans* which is involved in the signaling process of cellulose.

In contrast, deletion of *cltA* did not result in reduced biomass accumulation and cellulase activity in the presence of cellobiose, but introduction of CltA into *S. cerevisiae* conferred growth in the presence of different concentrations of cellobiose. These results indicate that CltA (formerly XtrG) is a cellobiose transporter and this is the first time that a cellobiose-specific transporter has been identified in *A. nidulans*. The genome of *A. nidulans* encodes 357 MFS transporters and redundancy is very likely to exist between these transporters. This redundancy could compensate for the individual loss of CltA (growth not affected) and CltB (growth restored after 72 h), suggesting that other transporters exist with transceptor activities. Deletion of both CltA and CltB had a more severe impact on fungal growth in the presence of cellobiose, suggesting that these two proteins do play major roles in cellobiose signaling and uptake and may work together to ensure growth on cellobiose. An intriguing aspect of the biology of *A. nidulans* CltA is why it is also induced in the presence of xylose when it actually is a cellobiose transporter. It is possible that cellobiose transporters could also transport xylooligosaccharides or that the main transcriptional activator of genes encoding proteins required for xylose and xylan metabolism, XlnR (on which CltA was shown to be dependent) can also induce genes encoding cellobiose transporters. Actually, it has already been demonstrated that *N. crassa* CDT-2 is able to transport both cellodextrins and xylodextrins [54]. Indeed, in *A. niger*, *cbhA,* and *cbhB*, encoding cellobiohydrolases which catalyze the depolymerization of cellulose were shown to be expressed at much higher levels in the presence of xylose and xylan than when compared to sophorose and cellulose [55]. Furthermore, when grown in the presence of xylan or cellulose, *A. nidulans* always secretes both cellulases and xylanases (data not shown). This is probably due to the fact that cellulose and hemicelluloses are always found together as they make up the plant cell wall.

Another characteristic of sugar transporters of the MFS is that they very often accept multiple monosaccharides and are, therefore, capable of transporting different sugars into the fungal cells; for example, XtrD transports various monosaccharides [33]. Previously, four glucose transporters were identified in *A. nidulans* (HxtB-HxtE) and we decided to verify if they could be involved in xylose transport. Deletion of *hxtB*, and to some extent *hxtE*, resulted in significantly reduced growth in the presence of high (2 % w/v) concentrations of xylose after 48 h. HxtB (also named MstC) has previously been shown to be a high-affinity glucose transporter which also appears to be able to translocate mannose, galactose, fructose, and xylose [56]. The expression of *hxtB* is not induced in the presence of glucose but rather in the absence of it, and this gene is also subject to CreA-mediated carbon catabolite repression [56]. This difference in growth and gene expression between high and low concentrations of xylose may therefore be explained by the high-affinity uptake system in which HxtB plays a role or due to the different time points at which both assays were carried out. Furthermore, gene transcription does not necessarily reflect protein secretion and growth. Growth on glucose was not affected, probably, because HxtB is a high affinity glucose transporter which is expressed when glucose is present in low concentrations. Glucose uptake in the presence of high concentrations of this sugar occurs via low affinity transporters such as MstE, which is induced by glucose in *A. nidulans* [57]. Furthermore, deletion of *hxtB* resulted in decreased affinity for and decreased transport capacity of xylose, indicating that HxtB is also a xylose transporter in addition to being a glucose transporter. In agreement, when HxtB::GFP was introduced into *S. cerevisiae*, where it located to the plasma membrane, it conferred growth of *S. cerevisiae* in the presence of different concentrations of xylose and was capable of successfully transporting xylose into the yeast cell. Furthermore, the *S. cerevisiae* HxtB::GFP strain produced ethanol when growing in xylose-rich media. This work, therefore, identified an *A. nidulans* transporter which in addition to taking up glucose was also efficient at transporting xylose.

Glucose is the preferred carbon source for most microorganisms as it provides rapid energy for survival and niche colonization. Hence, most fungi, including *A. nidulans* are specialized in taking up glucose as soon as it is detected in the environment through high and low affinity uptake systems. As shown in this work, *A. nidulans* transporters have preferentiality for glucose but when this sugar is not available, switch to transporting other sugars, such as xylose, arabinose, or galactose. In addition to the search for transporters which can also translocate pentose or cellodextrins, the focus of research should be directed to the molecular engineering of individual transporters to render them “blind” to glucose and increase affinity and specificity for alternative, non-glucose sugars as was done by [23, 58]. In addition, yeast strains, which already harbor heterologous introduced transporters, can be genetically modified through directed evolution to improve growth in the presence of pentose sugars or cellodextrins [33]. At the same time, introducing components required for the efficient transport and metabolism of various different sugars into *S. cerevisiae*, thus allowing the co-fermentation of multiple carbohydrates, has also proved to be a successful strategy [59, 60]. Furthermore, although the genome of *A. nidulans* (and other filamentous fungi) encodes a multitude of MFS sugar transporters, they have scarcely been characterized and further studies, including those on carbon source sensing and signaling, are required to confirm or reject the above proposed hypothesis. This work identified a cellobiose transporter and a potential cellobiose transceptor in *A. nidulans*, a role which has also been associated with cellobiose transporters in *N. crassa* and *T. reesei*. Furthermore, this study provided further characterization of a glucose/xylose transporter. Taken together, this work provides a preliminary screening and characterization of MFS transporters in *A. nidulans* and lays a basis for further exploration of sugar sensing and transport in industrially relevant fungi.

## Conclusions

The knowledge on sugar transport in fungi is very limited, although it presents a key step in the conversion of lignocellulosic biomass to biofuels. In this work, a cellobiose transporter, a xylose transporter, and a putative cellobiose transceptor were identified and characterized in *A. nidulans*. This is the first time that a sensory role for a sugar has been associated to a protein in this fungus. This study, therefore, highlights the importance of continuously screening fungal genomes for transporter-encoding genes and in addition, functionally characterizing these proteins. Furthermore, another drawback in the second-generation bioethanol production is the presence of glucose which represses proteins required for the utilization of alternative carbon sources. The identified xylose transporter is also a major glucose transporter, highlighting the preference of *A. nidulans* for this sugar. Furthermore, targeted molecular protein engineering could render these transporters more specific for non-glucose carbon sources. This work, therefore, presents a preliminary basis for further studies which would characterize and engineer known and novel transporters with the aim to introduce them into fermenting yeast strains to successfully convert a large amount of plant cell wall sugars into ethanol.

## Methods

### Strains, media, and culture methods

A list of all the strains used in this work is given in Table 2. All *A. nidulans* strains were grown at 37 °C in either liquid (without agar) or solid (with 20 g/l agar) minimal medium [MM: 1 % (w/v) carbon source, 50 ml of a 20× salt solution (120 g/l NaNO_3_, 10.4 g/l KCl, 30 g/l KH_2_PO_4_, 10.4 g/l MgSO_4_), 1 ml of 5× trace elements (22.0 g/l ZnSO_4_, 11 g/l boric acid, 5 g/l MnCl_2_, 5 g/l FeSO_4_, 1.6 g/l CoCl_2_, 1.6 g/l CuSO_4_, 1.1 g/l (NH_4_)_2_MoO_4_, 50 g/l ethylenediaminetetraacetic acid (EDTA)] and adjusted to pH 6.5 with NaOH. Depending on the auxotrophy of the strain, uridine (1.2 g/l), uracil (1.2 g/l) or pyridoxine (0.005 mg/μl) were added. All *S. cerevisiae* strains were grown at 30 °C in liquid (no agar) or solid (20 g/l agar) YNB medium (7 g/l yeast nitrogen base without amino acids, 0.05 g/l histidine, 0.1 g/l lysine, 0.1 g/l leucine, 0.1 g/l tryptophan, 0.1 g/l uridine, and 0.1 g/l uracil). All reagents were obtained from Sigma Aldrich (St. Louis, MO, USA), except where stated.

**Table 2:** Strains and plasmids used in this work

Strains/plasmids	Genotype	Reference
*S. cerevisiae*		
EBY.VW4000	CEN.PK2-1C *hxt13Δ::loxP hxt15Δ::loxP hxt16Δ::loxP hxt14Δ::loxP hxt12Δ::loxP hxt9Δ::loxP hxt11Δ::loxP hxt10Δ::loxP hxt8Δ::loxP hxt514Δ::loxP hxt2Δ::loxP hxt367Δ::loxP gal2 Δ stl1Δ::loxP agt1Δ::loxP ydl247wΔ::loxP yjr160cΔ::loxP*	[48]
SC9721	MATa *his 3-D200 URA 3-52 leu2D1 lys 2D202 trp 1D63*	FGSC
EBY.VW4000 +pRH195m +pRH274	EBYVW4000 pRH195 pRH274	[33]
hxtBGFP EBY.VW4000	EBYVW4000 pRH195 *hxtB* pRH274	This work
xtrF::GFP EBY.VW4000	EBYVW4000 pRH195 *xtrF* pRH274	This work
xtrG::GFP EBY.VW4000	EBYVW4000 pRH195 *xtrG* pRH274	This work
xtrH::GFP EBY.VW4000	EBYVW4000 pRH195 *xtrH* pRH274	This work
cltB::GFP EBY.VW4000	EBYVW4000 pRH195 *cltB* pRH274	This work
SC9721 cltA::GFP gh1-1	SC9721 pRH195 *cltA* pGH1	This work
SC9721 cltB::GFP gh1-1	SC9721 pRH195 *cltB* pGH1	This work
SC9721_pGH1	SC9721 pGH1	This work
SC9721_pCDT-1 gh1-1	SC9721 pCDT-1 pGH1	This work
*A. nidulans*		
TN02A3	*pyroA4 pyrG89; chaA1;* Δ*nKuA::argB*	[70]
HxtB::GFP TN02A3	pyrG89; pyroA4; Δnku70::argB; hxtB::GFP::pyrG	This work
HxtE::GFP TN02A3	pyrG89; pyroA4; Δnku70::argB; hxtE::GFP::pyrG	This work
* ΔxtrF*	*pyroA4 pyrG89; chaA1*; Δ*nKuA::argB; ΔxtrF::pyrG*	This work
* ΔxtrG/ΔcltA*	*pyroA4 pyrG89; chaA1*; Δ*nKuA::argB; ΔxtrG::pyrG*	This work
* ΔxtrH*	*pyroA4 pyrG89; chaA1*; Δ*nKuA::argB; ΔxtrH::pyrG*	This work
* ΔcltB*	*pyroA4 pyrG89; chaA1*; Δ*nKuA::argB; ΔcltB::pyroA4*	This work
* ΔhxtB*	*pyroA4 pyrG89; chaA1*; Δ*nKuA::argB; ΔhxtB::pyroA4*	[46]
* ΔhxtE*	*pyroA4 pyrG89; chaA1*; Δ*nKuA::argB; ΔhxtE::pyroA4*	[46]
* ΔcltA ΔcltB*	*pyroA4 pyrG89; chaA1*; Δ*nKuA::argB; ΔcltA::pyrG89; ΔcltB::pyroA4*	This work
GR5	*wA1 pyroA1pyrG89*	FGSC
* ΔcltB::cltB* ^+^	*ΔcltB::cltB* ^+^ *::pyrG* ^+^ *pyrG89*	This work
* oCltB3*	*wA1 pyroA1 pyrG89 cltB3::gfp::pyrG* ^+^	This work
Plasmids		
pRH195^a^	pBluescript II SK+, *TRP1, CEN6, ARSH4*+ *PHXT7-XKS1-THXT7*	[24]
pRH274	pBluescript II SK+, *URA3, CEN6, ARSH4* + P*PGK*1-*XYL1*-T*PGK*1; P*ADH*1-*XYL2*-T*ADH*1; P*HXT*7-*XKS1*-T*HXT*7	[66]
pRS426	*ampR lacZ* URA3	[65]
pCDA21	*Zeo::pyr ampR*	[71]
pGH1-1	pRS425 *PGK1p-gh1-1-CYC1t*	[26]
pCDT-1	pRS426 *PGK1p-cdt-1-CYC1t*	[26]

^a^The original vector pRH195 carries the XKS1 gene which was released after digestion with Spe*I* and Sal*I*. The resultant vector without the XKS1 gene was used in this work for compl ementation assays

### Construction of *Aspergillus nidulans* null mutants

Standard genetic techniques for *A. nidulans* strain constructions, transformations, and DNA manipulations were done according to [61]. PCR reactions were performed using Phusion High-Fidelity DNA polymerase (New England Biolabs) or *TaKaRa Ex Taq DNA Polymerase* (Clontech USA). A list of all primer pairs can be found in Additional file 4. The gene knock-out strains *ΔxtrF*, *ΔxtrG*, *ΔxtrH,* and *ΔcltB* (AN0332, AN8347, AN9173 and AN2814, respectively) were obtained through replacing each gene with a prototrophic marker gene. Gene replacement cassettes were generated by in vivo recombination in *S. cerevisiae* as previously described by [62]. Briefly, the 5′ UTR of each target gene was PCR amplified using specific primers: *xtrF* (primers P1 and P2), *xtrG* (primers P7 and P8), *xtrH* (primers P13 and P14), and *cltB* (primers P27 and P28). Similarly, the 3′UTR regions of each gene were amplified by PCR: *xtrF* (primers P3 and P4), *xtrG* (primers P9 and P10), *xtrH* (primers P15 and P16), and *cltB* (primers P29 and P30). Pyridoxine or uridine/uracil were used as prototrophic markers, and their respective genes (*pyroA* and *pyrG*) were amplified by PCR from plasmids pAFpyro (primers P37 and P38) and pCDA21 (primers P35 and P36), respectively (Table 2). The individual gene fragments (5′ and 3′ UTRs and prototrophic marker gene) were transformed, together with plasmid pRS426, which was linearized with *EcoR*I and *BamH*I, into *S. cerevisiae* SC9721 using the lithium acetate method [63]. Positive *S. cerevisiae* transformation candidates were grown in YNB-URA medium, before gDNA was extracted and PCRs were run to confirm the correct construction. The cassettes were then PCR-amplified from *S. cerevisiae* genomic DNA, purified and used to transform *A. nidulans* TN02A3 strain, according to [64]. Positive *A. nidulans* transformation candidates were selected and purified through three rounds of growth on plates and gDNA was extracted. Gene deletions were confirmed by Southern blots (Additional file 5).

To construct the complemented strain Δ*cltB::cltB*^+^, the complementing cassette containing the 5′ UTR region plus the cltB gene and the 3′ UTR region was amplified by PCR from *A. nidulans* genomic DNA using specific primers (P27 and P30). The *A. nidulans ΔcltB Ku80*^+^ mutant was co-transformed with pCDA21 plasmid and the *cltB*^+^ complementing cassette. Positive *A. nidulans* complemented candidates were selected and purified through three rounds of growth on plates, gDNA was extracted, and the candidates were confirmed by PCR (Additional file 6).

### *Construction of Aspergillus nidulans GFP*- *tagged strains*

All *A. nidulans* GFP-tagged strains were constructed as described in the previous section (“Construction of *A. nidulans* null mutants”) with the exception that genes were not replaced by prototrophic markers but were instead C-terminally tagged with GFP. The selective marker gene *pyrG* was also introduced. A list of all primers used for strain constructions can be found in Additional file 4. The xtrF-H, hxtB and hxtE genes were amplified by PCR using primers P5/P6, P11/P12, P17/P18, P42/P43, and P48/P49, respectively. The 3′ UTRs of genes *xtrF*-*H*, *hxtB,* and *hxtE* constructions were amplified by PCR using primers P3/P4 (*xtrF*), P9/P10 (*xtrG*), P15/P16 (*xtrH*), P30/P32 (*cltB*), P44/P45 (*hxtB*), and P50/P51 (*hxtE*). The *gfp* gene was separated from the target gene by four additional codon triplets that after translation produce a four amino acid residue linker (glycine–threonine–arginine–glycine) region termed Spacer-GFP [65]. To allow fusion of GFP to our protein of interest, the stop codon of the gene ORF was removed when designing the primers. The GFP was amplified from the pMCB17apx plasmid (kindly provided by Vladimir P. Efimov) with primers P39/P40. The *pyrG* gene was amplified as described above. GFP-tagged gene constructions were confirmed by PCR in *A. nidulans*.

In addition, the *cltB* overexpression strain (GR5 CltB::GFP strain) was constructed in the *A. nidulans* GR5 background, because this strain allows multiple non-homologous ectopic integrations. Again, the *A. nidulans* GFP-tagged strains were constructed as described in the previous section (“Construction of *A. nidulans* null mutants”) with the exception that genes were not replaced by prototrophic markers but were instead C-terminally tagged with GFP and the selective marker gene *pyrG* was also introduced. The cltB gene (primers P27/P31) and the 3′ UTR (primers P30/P32) were amplified by PCR. The multiple integrations of CltB::GFP cassette were confirmed by Southern blot (Additional file 1).

For all constructions above described, the *gfp* gene was separated from the target gene by four additional codon triplets that after translation produce a four amino acid residue linker (glycine–threonine–arginine–glycine) region termed Spacer-GFP [65]. To allow fusion of GFP to our protein of interest, the stop codon of the gene ORF was removed when designing the primers. The GFP was amplified from the pMCB17apx plasmid (kindly provided by Vladimir P. Efimov) with primers P39/P40. The *pyrG* gene was amplified as described above. GFP-tagged gene constructions were confirmed by PCR in *A. nidulans* or southern blot. The GFP was amplified from the pMCB17apx plasmid (kindly provided by Vladimir P. Efimov) with primers P39/P40. The *pyrG* gene was amplified as described above. For *xtrF*-*H*, *hxtB* and *hxtE* mutants, GFP-tagged gene constructions were confirmed by PCR in *A. nidulans*.

### Construction of *Saccharomyces cerevisiae* strains

Strain EBY.VW4000 (Table 2) was used for the *Saccharomyces cerevisiae* complementation assays [48]. A list of all primers can be found in Additional file 4. The *xtrF*-*H*, *cltB,* and *hxtB* ORFs were amplified by PCR from cDNA obtained from *A. nidulans* strains using primers P19/P20 (*xtrF*), P21/P22 (*xtrG*), P23/P24 (*xtrH*), P33/P34 (*cltB*), and P46/P47 (*hxtB*), respectively. Plasmid pRH195 was double digested with *Spe*I and *Sal*I for linearization and release of the *XKS1* gene (generating the pRH195 m). For in vivo recombination, plasmid pRH195 m was transformed into *S. cerevisiae E*BY.VW4000, which already contained plasmid pRH274 [33], together with all the PCR-amplified sugar transporters and GFP fragments using the lithium acetate method [63]. The *gfp* gene was amplified from plasmid pMCB17apx using primers P25/P26. *S. cerevisiae* EBY.VW4000 is unable to metabolize xylose and in addition to being transformed with the *A. nidulans* transporter-encoding genes, it was also transformed with genes encoding enzymes of the xylose metabolic pathway. *Saccharomyces stipitis* xylose reductase (XR) and xylose dehydrogenase (XDH) as well as *S. cerevisiae* xylulose kinase (XK) were introduced in EBY.VW4000 via plasmid pRH274 (Table 2), where the three enzyme-encoding genes were placed under the control of the *PGK1*, *ADH1,* and *HXT7* constitutive promoters, respectively [66]. Transformants were selected for tryptophan and uridine prototrophy on solid YNB lacking both tryptophan and uridine and supplemented with 2 % maltose.

*Saccharomyces cerevisiae* SC9721 strain was used to construct the yeast strains expressing the cellobiose transporters *cltA* and *cltB*. The SC9721 strain was first transformed with the pGH1 plasmid which contains the β-glucosidase-encoding gene *gh1*-*1* from *N. crassa* [26]. The *cltA* and *cltB* cellobiose transporter genes were amplified from cDNA of *A. nidulans* using primers P52/P22 (*cltA*) and P53/P34 (*cltB*). Plasmid pRH195 m was used to transform *S. cerevisiae* with the respective transporter genes. Furthermore, *S. cerevisiae* strain SC9721 was also transformed with plasmid pCDT-1, containing the already characterized *N. crassa cdt*-*1* cellobiose transporter-encoding gene, which was used as a positive control in ours assays [12]. All *S. cerevisiae* transformations were carried out using the lithium acetate method [63] and strain constructions were confirmed by PCR.

### gDNA extraction from *A. nidulans and S. cerevisiae*

Genomic DNA extractions of *A. nidulans* and *S. cerevisiae* were performed according to [67] and [63].

### Microscopy

*A. nidulans* strains HxtB::GFP, HxtE::GFP, XtrF::GFP, XtrG::GFP, XtrH::GFP, and CltB::GFP were grown from spores in 3 ml of MM containing 0.1 and 1 % xylose for 10, 15, 20, and 24 at 30 °C in a small Petri dish containing a microscopy cover slip. The oCltB3::GFP strain was previously inoculated 16 h at 30 °C in a small Petri dish containing a microscopy coverslip and 3 mL of MM supplemented with 1 % fructose as a carbon source. After 16 h, the germlings were washed with 1X PBS and transferred to 0.5 and 1 % cellobiose for 4 or 8 h. Coverslips were washed with 1× PBS (137 mM sodium chloride, 2.7 mM potassium chloride, 10 mM disodium hydrogen phosphate, 1.8 mM potassium dihydrogen phosphate) and viewed under the microscope. *S. cerevisiae* EBY.VW400 strain harboring the *hxtB*, *xtrF*-*H,* and *cltB* tagged to GFP constructions was grown 48 h in 0.5 ml of liquid YNB-trp-ura medium supplemented with 2 % maltose for 24 h at 30 °C in a 24-wells plate. Cells were washed with PBS and viewed under the microscope. All slides were viewed with a Carl Zeiss (Jena, Germany) microscope using the 100× magnification oil immersion objective lens (EC Plan-Neofluar, NA 1.3) equipped with a 100-W HBO mercury lamp epifluorescence module. Phase contrast brightfield and fluorescent images were taken with an AxioCam camera (Carl Zeiss), and images were processed using the AxioVision software version 3.1 and saved as TIFF files. Further processing was performed using Adobe Photoshop 7.0 (Adobe Systems Incorporated, CA).

### Dry weight measurement

A total of 5 × 10^7^ spores of *A. nidulans* wild-type (TN02A3) and mutant strains (*ΔxtrF*-*H* and *ΔhxtB*) were inoculated in 50 ml of liquid minimal medium supplemented with 1 % glucose, 2 % xylose or 0.2 % xylose. 2.5 × 10^7^ spores of *A. nidulans* wild-type (TN02A3) and mutant strains (Δ*cltA*, *ΔcltB and ΔcltA ΔcltB)* were inoculated in 50 mL of liquid minimal medium supplemented with 1 % glucose or 1 % cellobiose. Strains were grown for 24 and 48 h in xylose-rich media and for 48 and 72 h in cellobiose-rich media at 37 °C, 180 rpm. Mycelia were harvested by vacuum filtration, snap-frozen in liquid N_2_, freeze-dried and subsequently weighed.

### RNA extraction and real-time PCR reactions

To measure the expression of *xtrF*-*H*, a total of 10^7^ spores from the *A. nidulans* wild-type, *creAd30* or Δ*xlnR* strains were inoculated in 50 ml of liquid MM containing 1 % fructose for 16 h at 37 °C, 180 rpm. Mycelia were washed with sterile water and transferred to MM supplemented with 1 % xylose or 1 % xylose and 1 % glucose for 6, 12 and 24 h at 37 °C, 180 rpm. Alternatively, 10^7^ spores from the wild-type *A. nidulans* strain was inoculated in 50 ml of liquid MM supplemented with 1 % glucose, 1 % sorbitol, 1 % xylose, 1 % fructose, 1 % maltose, 1 % galactose, and 1 % mannose at 37 °C, 180 rpm for 8 or 16 h. All mycelia were harvested by vacuum filtration, snap-frozen in liquid N_2_, and stored at −80 °C.

To measure the expression of *cltA* and *cltB*, a total of 10^7^ spores from the *A. nidulans* wild-type strain were inoculated in 50 ml of liquid MM containing 1 % fructose for 16 h at 37 °C, 180 rpm. Mycelia were washed with sterile water and transferred to MM supplemented with 1 % cellobiose for additional 1, 2, and 4 h at 37 °C, 180 rpm. Mycelia were harvested by vacuum filtration, snap frozen in liquid N_2_, and stored at −80 °C. cltA and cltB

Mycelia were ground to a fine powder under liquid N_2_, and RNA was extracted using Trizol (Invitrogen, Carlsbad, CA, USA), according to manufacturer’s instructions. The quality of the RNA (10 μg) was checked by running them through the Bioanalyzer. RNA samples were DNAse-treated as previously described by [67], purified with the RNeasy^®^ Mini Kit (Qiagen, Valencia, CA, USA) and quantified on the NanoDrop^®^ 2000 (Thermo Scientific) machine. RNA integrity was confirmed using the Bioanalyser Nano Kit (Agilent Technologies) and the Agilent Bioanalyser 2100, using an RIM value of 6.0 as a threshold.

RNA was then reverse transcribed to cDNA using the Superscript III Reverse transcriptase kit (Invitrogen), according to manufacturer’s instructions. All RT-qPCR reactions were performed using the ABI 7500 Fast Real-Time PCR System (Applied Biosystems, Foster City, CA, USA) and the SYBR Green PCR Master Mix kit (Applied Biosystems), according to manufacturer’s instructions. Reactions and calculations were performed as previously described [68]. All primers are listed in Additional file 4.

### Xylose uptake assay

Xylose uptake rates were measured by monitoring the incorporation of D-[U-^14^C] xylose [289.0 mCi/mmol (10.693 GBq)/mmol] (Perkin Elmer Life Sciences) into germinating conidia in the presence of various d-xylose concentrations according to [33] with modifications. A total of 1.2 × 10^9^ Δ*hxtB* conidia were inoculated in 600 ml MM containing 1 % glycerol (w/v) for 5 h at 37 °C, 180 rpm. Swollen conidia were harvested by vacuum filtration through nitrocellulose filters (Fisherbrand) and washed twice with ice-cold water. Conidia were re-supended in water to get a concentration of 2.5 × 10^7^ conidia/250 μl. A total of 2.5 × 10^7^ spores were inoculated with different concentrations of D-xylose (0.1–100 mM) in 1.5 ml tubes together with 1 μl of radiolabelled ^14^C-xylose (0.2 μCi) and incubated at room temperature for 30–60 s. Xylose uptake was stopped by adding 1.5 ml ice-cold water and conidia were immediately harvested by vacuum filtration through nitrocellulose filters. Conidia were washed again two times with 1.5 ml ice-cold water.

*Saccharomyces cerevisiae* HxtB::GFP EBY.VW400 strain was inoculated in 300 ml of YNB medium supplemented with 2 % maltose until they reached the exponential growth phase (OD_600nm_ of 0.6). Yeast cells were pelleted by centrifugation at 4000 rpm for 5 min, washed twice with 50 ml ice-cold water, and then re-suspended in 4.5 ml of ice-cold water. 40 μl of this cell suspension were transferred to 1.5 ml Eppendorf tubes which were then incubated at 30 °C for 5 min for temperature equilibration. 10 μl of different concentrations of xylose (0.1 to 100 mM xylose) and 0.2 μCi of ^14^C-xylose were added to the yeast cells. Xylose uptake was allowed to proceed for 10 s through vigorous vortexing before the reaction was stopped through the addition of 1.5 ml ice-cold water. Cells were harvested by vacuum filtration through nitrocellulose filters and washed two times with ice-cold water.

All nitrocellulose filters containing the fungal cells were transferred to 3 ml of ScintiSafeTM Econo1 scintillation liquid (Fisher Scientific), and the D-[U-^14^C] xylose taken up by the cells was measured using the Tri-Carb^®^ 2100TR Liquid Scintillation Counter.

### Assaying extracellular xylose concentrations

The EBY.VW4000 + pRH195 m + pRH274 (control) and the HxtB::GFP yeast strains were inoculated (initial OD_600_ 0.5) in 50 ml YNB-trp-ura medium supplemented with 1 % (w/v) xylose at 30 °C, 150 rpm for 300 h. At each time point, 2.0 ml of the culture was collected, centrifuged, and the supernatants were stored at −80 °C. The xylose concentration in the supernatants was measured using the d-xylose assay kit (Megazyme) following manufacturer’s instructions. Absorbance was measured at 340 nm in a 96-well polystyrene plate (Corning) using the SpectraMax I3 spectrometer (molecular devices).

### Assaying extracellular ethanol concentrations

The EBY.VW4000 + pRH195 m + pRH274 (control) and HxtB::GFP yeast strains were inoculated (initial OD_600_ 0.5) in 50 ml YNB-trp-ura medium supplemented with 1 % (w/v) xylose at 30 °C, 150 rpm for 300 h. At each time point, 2.0 ml of the culture was collected, centrifuged, and the supernatants were stored at −80 °C. Ethanol production was determined by measuring the absorption of NADH at 340 nm as previously described [69] with modifications. Reactions were started through mixing 100 µl assay buffer (50 mM pyrophosphate, 50 mM semicarbazide, and 20 mM glycine, pH 8.8) with 0.643 mM NAD^+^, 5 U alcohol dehydrogenase and 10 µl sample supernatant in a 96-well polystyrene plate (Corning). Samples were incubated at 30 °C for 5 min, and then, the ethanol concentration was measured at 340 nm using the SpectraMax I3 spectrometer (Molecular devices).

### Growth of *Saccharomyces cerevisiae* strains on solid medium

*S. cerevisiae* strains were inoculated in 50 ml YNB medium supplemented with 2 % maltose or 1 % glucose for 24 h at 30 °C, 150 rpm until an OD_600nm_ of 0.1. Yeast cells were centrifuged at 4.000 rpm for 5 min, washed two times with water, and re-suspended in water to a final concentration of 1.0 at OD_600nm_. A serial dilution of 1:10 of the yeast cells was made, and 5 μl of the cell suspensions were spotted on plates containing 1 % glucose, 0.1, 1 or 2 % cellobiose. Plates were incubated at 30 °C for 168 h.

### Yeast growth rates

The SC9721 cltA::GFP gh1-1, SC9721 cltB::GFP gh1-1 and SC9721 pCDT-1 gh1-1 strains were grown in YNB medium supplemented with 1 % glucose for 24 h at 30 °C, 150 rpm. The OD at 600 nm was measured; the cell cultures were diluted to OD_600nm_ of 0.1 and transferred to 50 ml YNB medium supplemented with the respective carbon source (glucose 1 % or cellobiose 0.1–2 %) at 30 °C, 150 rpm for 144 h. The OD_600nm_ was measured periodically at the indicated time points.

### Cellulase assays

A total of 10^7^ spores from wild-type, *ΔcltA*, *ΔcltB* and *ΔcltA ΔcltB* mutant strains were inoculated in liquid MM supplemented with 1 % fructose at 37 °C, 180 rpm for 16 h. Mycelia were washed with sterile water and transferred to MM supplemented with 1 % Avicel™ for 5 days at 37 °C, 180 rpm. The supernatant was separated from the mycelia using miracloth. Cellulase (endo-1,4-β-glucanase) activity in the supernatants was measured using Azo-CM-Cellulose (Megazyme International, Bray, Ireland) as a substrate, according to manufacturer’s instructions.

## Additional files

10.1186/s13068-016-0611-1 Construction of an overexpressing CltB::GFP mutant strain. (A) Genomic DNA from *A. nidulans* wild-type GR5 and CltB::GFP transformant strains were isolated and cleaved with the enzyme *Pst*I; a 2.7-kb DNA fragment from the 5′-noncoding region plus the *cltB* gene was used as a hybridization probe. This fragment recognizes a single DNA band (about 5.0 kb) in the wild-type strain and a single DNA band (about 3.0 kb) in the CltB::GFP homologously integrated cassette. Different size of bands indicates the multiple integration of the GFP cassette. (B) Southern blot.
10.1186/s13068-016-0611-1 Enzymatic kinetics. (A) Eadie–Hofstee and (B) Lineweaver–Burk plots for the data of the Fig. 6c.
10.1186/s13068-016-0611-1 HxtE is target to the plasma membrane in *A. nidulans* in the presence of xylose. The *A. nidulans* HxtE::GFP strain was grown from conidia in minimal media supplemented with 0.1 % or 1 % of xylose for 10 h, 15 h, 20 h, and 24 h. DIC (differential interference contrast) was applied to view unstained hyphae.
10.1186/s13068-016-0611-1 Primers used in this work.
10.1186/s13068-016-0611-1 Genomic DNA from the *A. nidulans* wild-type, Δ*xtrG* (AN8347), Δ*xtrH* (AN9173), Δ*cltB* (AN2814) and the double Δ*cltA* Δ*cltB* strains was extracted and digested with different restriction enzymes to confirm the deletion strains. Diagram (A.) and Southern blot (B.) of the wild-type and *ΔxtrG* strains when digested with *Sac*I. A 1-kb DNA fragment from the *xtrG* 3′UTR (untranslated) region was used as a hybridization probe. The probe recognizes a single 10.0-kb band in the wild-type strain and a single 6.4-kb band in the Δ*xtrG* strain. Diagram (C.) and Southern blot (D.) of the wild-type and Δ*xtrH* strains when digested with *EcoR*I. A 1-kb DNA fragment from the *xtrH* 5′UTR (untranslated) region was used as a hybridization probe. The probe recognizes a single 3.4-kb band in the wild-type strain and a single 3.0-kb band in the Δ*xtrH* strain. Diagram (E.) and Southern blot (F.) of the wild-type and Δ*cltB* strains when digested with *Xba*I. A 1-kb DNA fragment from the *cltB* 5′UTR (untranslated) region was used as a hybridization probe. The probe recognizes a single 2.0-kb band in the wild-type strain and a single 3.3-kb band in the Δ*cltB* strain. Diagram (G.) and Southern blot (H.) of the wild-type and Δ*cltA* Δ*cltB* strains when digested with *Kpn*I. A 1-kb DNA fragment from the *cltB* 3′UTR (untranslated) region was used as a hybridization probe. The probe recognizes a single 2.0-kb band in the wild-type strain and a single 2.5-kb band in the Δ*xtrG* strain.
10.1186/s13068-016-0611-1 PCR confirmation of the ∆*cltB::cltB* ^+^ strain. Genomic DNA from *A. nidulans* wild-type, complementing strains *∆cltB::cltB* ^+^ (candidates 1, 2, and 3) and deletion strain *∆cltB* were isolated and used as template for PCR reactions. (A) Specific primers P70 and P31 were used to amplify a DNA fragment of about 3.1 kb. (B) Specific primers P70 and P37 were used to amplify a DNA fragment of about 3.0 kb. Lanes 1 and 7: C- negative control with no DNA as a template; lanes 2 and 8: genomic DNA from the *∆cltB::cltB* candidate 1; lanes 3 and 9: genomic DNA from the *∆cltB::cltB* candidate 2; lanes 4 and 10: genomic DNA from the *∆cltB::cltB* candidate 3; lanes 5 and 11: genomic DNA from the *∆cltB* deletion strain; lanes 6 and 12: genomic DNA from the wild-type strain. The * indicates the candidate containing the homologous integration of the complementing cassette and (C^−^) the negative control.

## Abbreviations

2G: second-generation bioethanol
BP: base pairs
cDNA: complementary DNA
CDT: cellodextrin transporter
CLT: cellobiose transporter
CMC: carboxymethylcellulose
EDTA: ethylenediaminetetraacetic acid
GFP: green fluorescent protein
HXT: hexose transporter
Kb: kilo bases
*K*_*m*_: Michaelis constant
M: molar
MFS: major facilitator superfamily
MM: minimal medium
Nm: nanometre
OD: optical densisty
ORF: open reading frame
PBS: phosphate-buffered saline
PCR: polymerase chain reaction
pyrG: orotidine-5′-phosphate decarboxylase gene
Pyro: pyridoxine gene
RT-qPCR: quantitative reverse transcription PCR
TRP: tryptophan
V_max_: maximum reaction velocity rate
XDH: xylitol dehydrogenase
XKS1: xylulose kinase
XR: xylose reductase
XTR: xylose transporter
YNB: yeast nitrogen base
