# Supplementary material for: Identification and characterization of putative xylose and cellobiose transporters in Aspergillus nidulans
Source: Biotechnol Biofuels. 2016 Sep 26;9:204. doi: 10.1186/s13068-016-0611-1 (PMC5037631; doi:10.1186/s13068-016-0611-1)
Supplement: Supplementary file 1 — 10.1186/s13068-016-0611-1 Construction of an overexpressing CltB::GFP mutant strain. (A) Genomic DNA from A. nidulans wild-type GR5 and CltB::GFP transformant strains were isolated and cleaved with the enzyme PstI; a 2.7-kb DNA fragment from the 5′-noncoding region plus the cltB gene was used as a hybridization probe. This fragment recognizes a single DNA band (about 5.0 kb) in the wild-type strain and a single DNA band (about 3.0 kb) in the CltB::GFP homologously integrated cassette. Different size of bands indicates the multiple integration of the GFP cassette. (B) Southern blot. [file 13068_2016_611_MOESM1_ESM.pdf]

A.

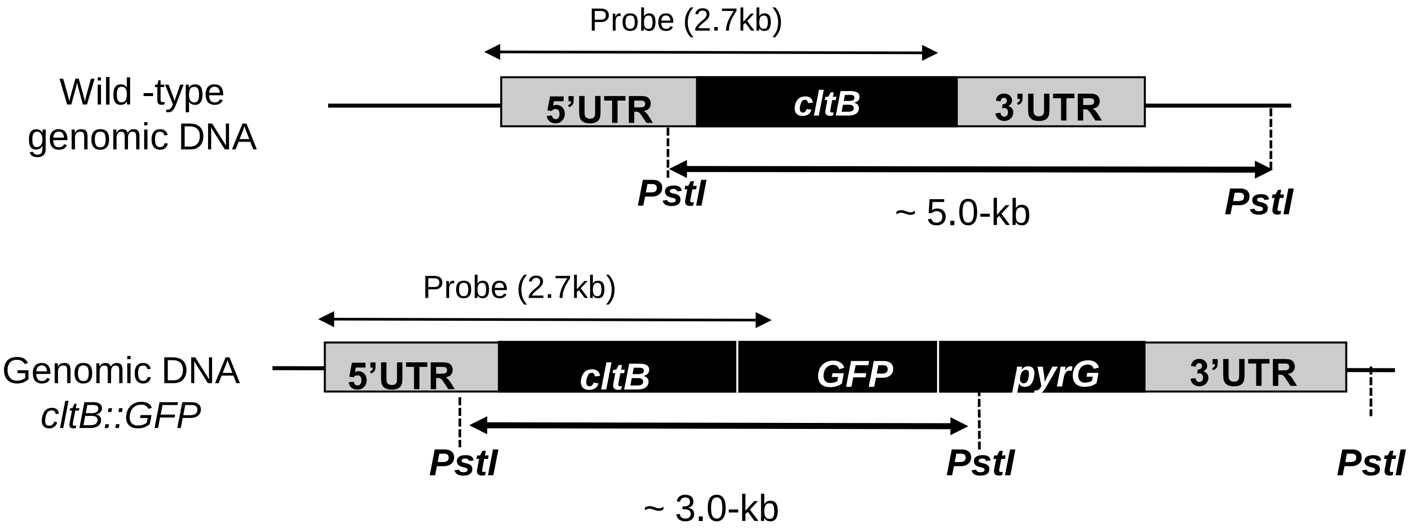

B.

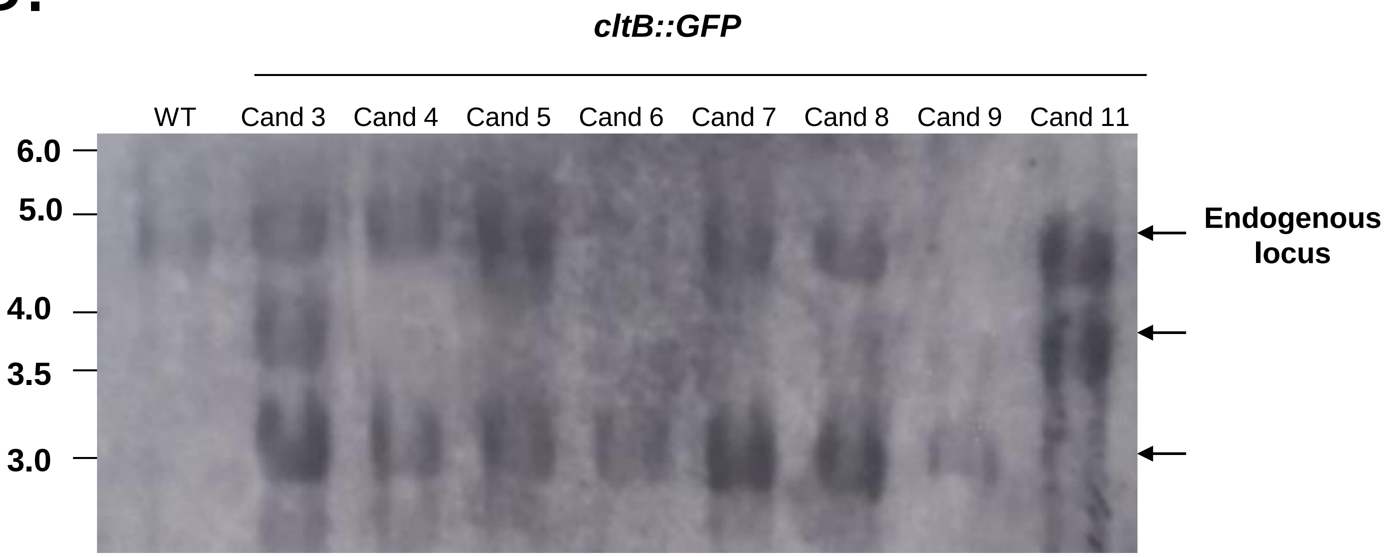

Additional file 1. Construction of an overexpressing CltB::GFP mutant strain. (A) Genomic DNA from *A. nidulans* wild type GR5 and *cltB::GFP* transformant strains were isolated and cleaved with the enzyme *Pst*I; a 2.7-kb DNA fragment from the 5'-noncoding region plus the *cltB* gene was used as a hybridization probe. This fragment recognizes a single DNA band (about 5.0-kb) in the wild-type strain and a single DNA band (about 3.0-kb) in the CltB::GFP homologously integrated cassete. Different size of bands indicate the multiple integration of the GFP cassete. (B) Southern blot.
