# Supplementary material for: Identification and characterization of putative xylose and cellobiose transporters in Aspergillus nidulans
Source: Biotechnol Biofuels. 2016 Sep 26;9:204. doi: 10.1186/s13068-016-0611-1 (PMC5037631; doi:10.1186/s13068-016-0611-1)
Supplement: Supplementary file 2 — 10.1186/s13068-016-0611-1 Enzymatic kinetics. (A) Eadie–Hofstee and (B) Lineweaver–Burk plots for the data of the Fig. 6c. [file 13068_2016_611_MOESM2_ESM.pdf]

A.

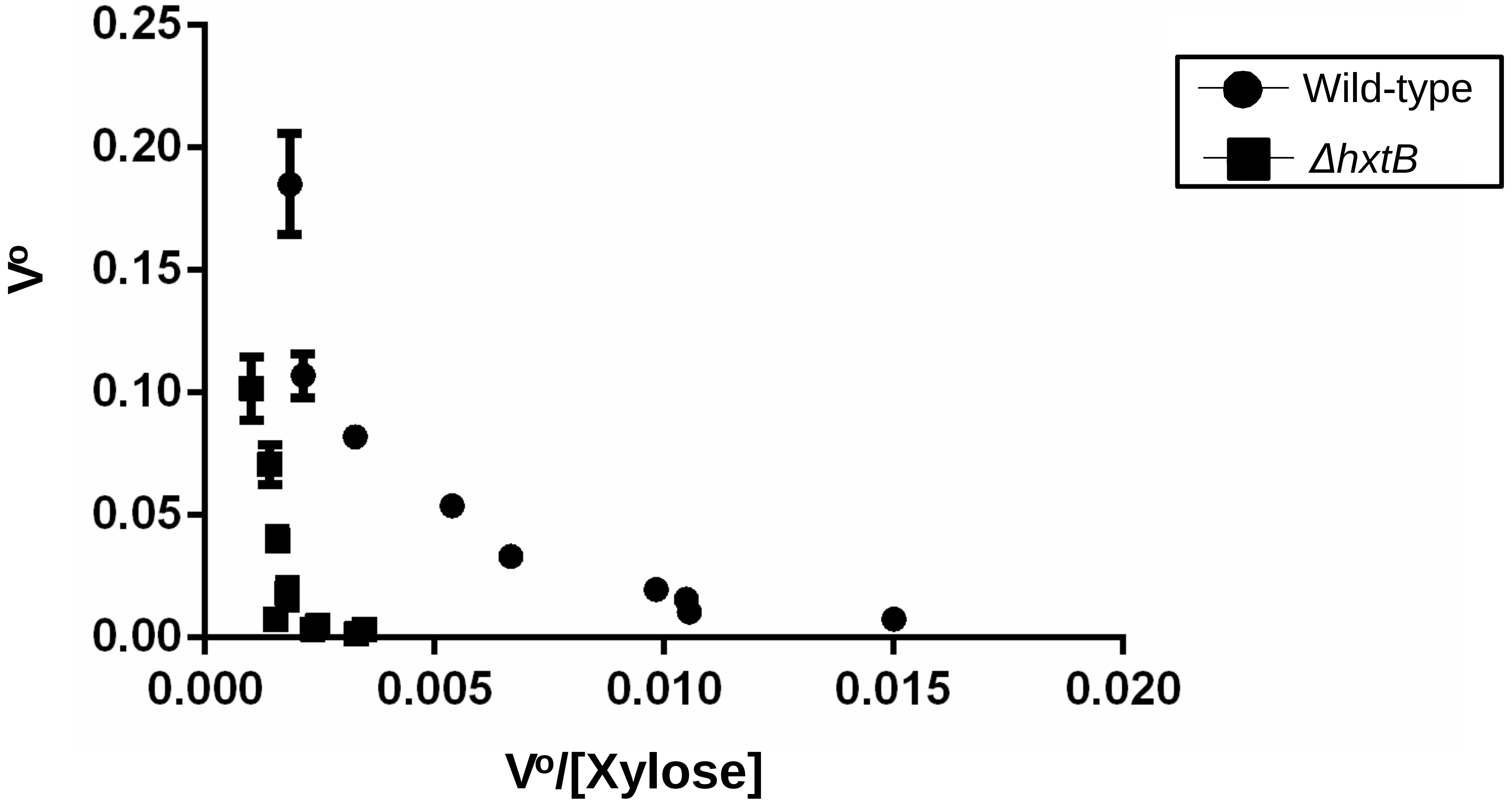

B.

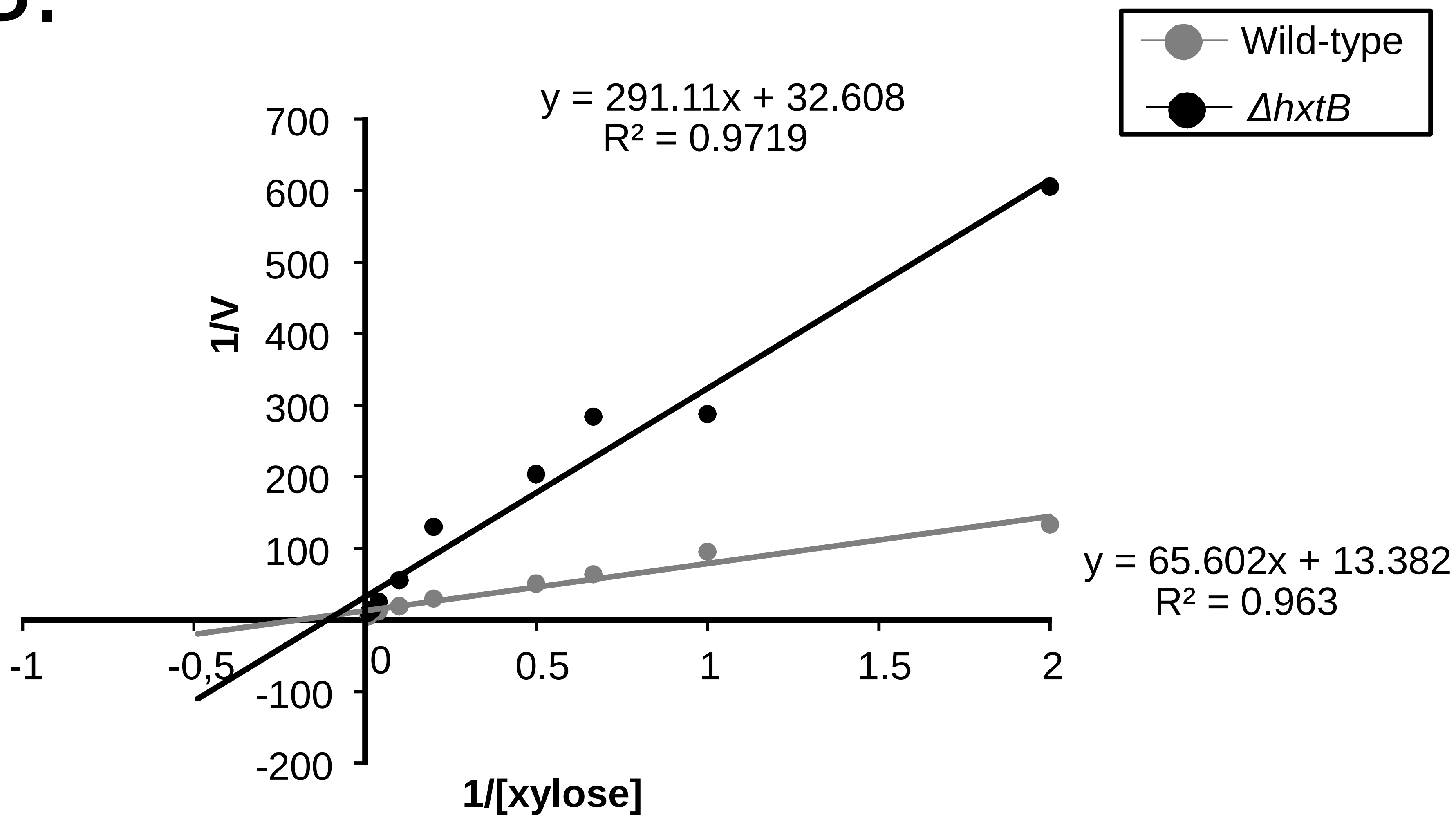

Additional file 2. Enzymatic kinetics. (A) Eadie-Hofstee and (B) Lineweaver-Burk plots for the data of the Figure 5C.
