# Supplementary material for: Identification and characterization of putative xylose and cellobiose transporters in Aspergillus nidulans
Source: Biotechnol Biofuels. 2016 Sep 26;9:204. doi: 10.1186/s13068-016-0611-1 (PMC5037631; doi:10.1186/s13068-016-0611-1)
Supplement: Supplementary file 3 — 10.1186/s13068-016-0611-1 HxtE is target to the plasma membrane in A. nidulans in the presence of xylose. The A. nidulans HxtE::GFP strain was grown from conidia in minimal media supplemented with 0.1 % or 1 % of xylose for 10 h, 15 h, 20 h, and 24 h. DIC (differential interference contrast) was applied to view unstained hyphae. [file 13068_2016_611_MOESM3_ESM.pdf]

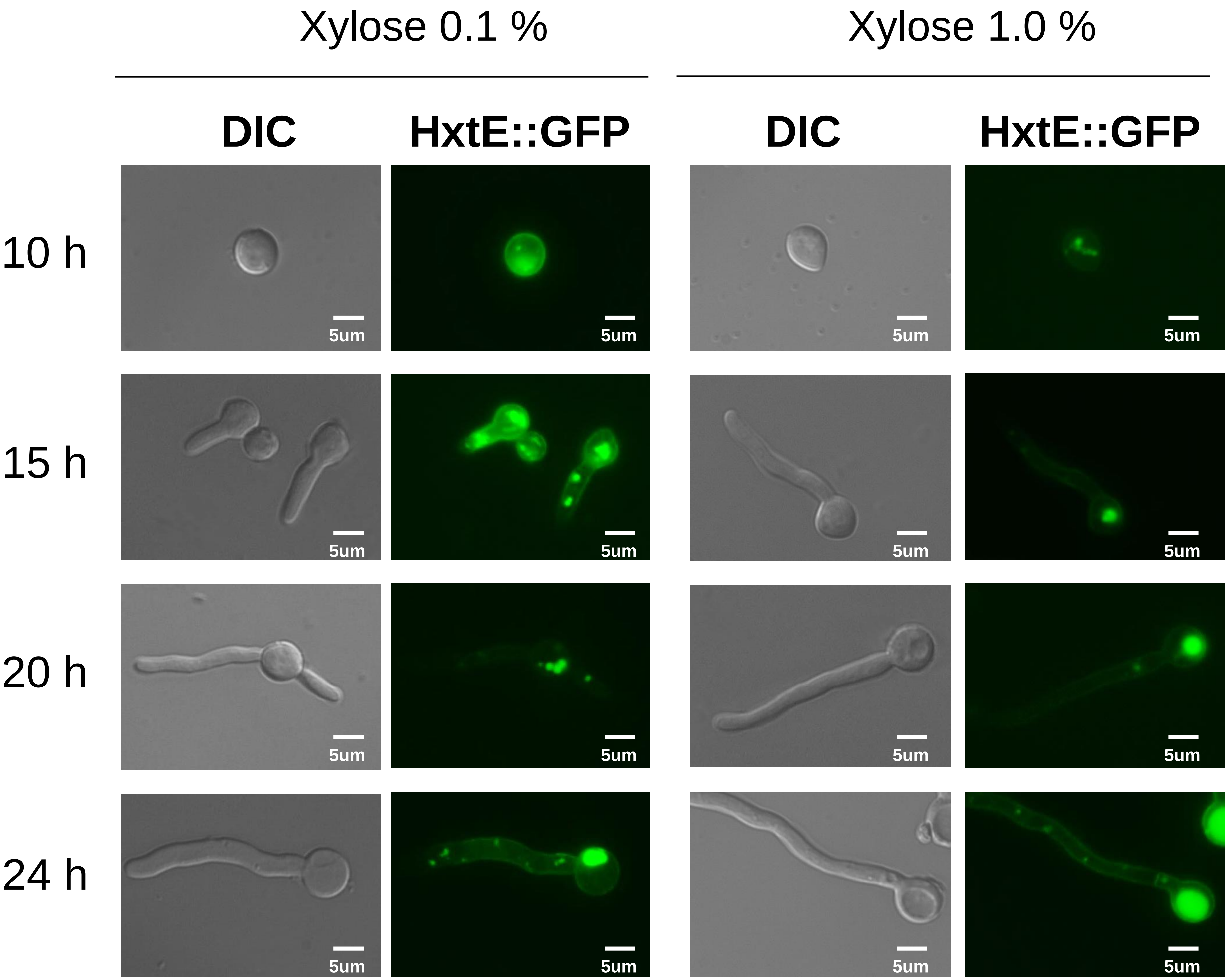
Xylose 0.1 %
Xylose 1.0 %
DIC
HxtE::GFP
DIC
HxtE::GFP
10 h
15 h
20 h
24 h
5um

Additional File 3. HxtE is targeted to the plasma membrane and small vacuoles in *A. nidulans* in the presence of xylose. The *A. nidulans* HxtE::GFP strain was grown from conidia in minimal media supplemented with 0.1% or 1% of xylose for 10 h, 15 h, 20 h, and 24 h. DIC (differential interference contrast) was applied to view unstained hyphae.
