# Supplementary material for: Identification and characterization of putative xylose and cellobiose transporters in Aspergillus nidulans
Source: Biotechnol Biofuels. 2016 Sep 26;9:204. doi: 10.1186/s13068-016-0611-1 (PMC5037631; doi:10.1186/s13068-016-0611-1)
Supplement: Supplementary file 4 — 10.1186/s13068-016-0611-1 Primers used in this work. [file 13068_2016_611_MOESM4_ESM.pdf]

Additional File 4. Primers used in this work

	Primers	Sequence (5´- 3´)
P1	xtrF 5F pRS426	GGTTTTCCCAGTCACGACGGCCAGTTGCTCGTCGGACAG
P2	xtrF 5R pyrG	GTGCCTCCTCTCAGACAGAATATTGAGGGTCGGCGAGGAAC
P3	xtrF 3F pyrG	GCATTGTTTGAGGCGAATTCTCTGAGGGATGCTTTATCAG
P4	xtrF 3R pRS426	CTCTCTGGAAACAGCTCAACATGGATAATGGTATAC
P5	xtrF ORF pRS426 F	GGTTTTCCCAGTCACGACGCACGCTTGGAATCCCTCTGG
P6	xtrF ORF_Spacer GFP R	GTTCTTCTCCTTTACTCATTCCCCGTGTTCCCTCTTTCATTGA TGGCCGAAG
P7	xtrG 5F pRS426	GGTTTTCCCAGTCACGACGTGGTAAGGACCCTTGTTGCC
P8	xtrG 5R pyrG	GCCTCCTCTCAGACAGAATGGCGCTATAGACGGATACGC
P9	xtrG 3F pyrG	GCATTGTTTGAGGCGAATTCTAGCGTAGGACCCATGCAAG
P10	xtrG 3R pRS426	CTCTCTGGAAACAGCGACGTACTCTGGACGCTCGC
P11	xtrG ORF pRS426 F	GGTTTTCCCAGTCACGACGGAGAAGGCAAAGAAGATATTGG
P12	xtrG ORF_Spacer GFP R	GTTCTTCTCCTTTACTCATTCCCCGTGTTCCCTCGGAATTCAA AGTCCTTC
P13	xtrH 5F pRS426	GGTTTTCCCAGTCACGACGCTAATCTCAGATCGATGAATC
P14	xtrH 5R pyrG	GCCTCCTCTCAGACAGAATCCTGACCAGCAGATTGCACTC
P15	xtrH 3F pyrG	GCATTGTTTGAGGCGAATTCGTGTCATCGTTTGTGTAAGC
P16	xtrH 3R pRS426	CTCTCTGGAAACAGCCATTAGCGCCAAGGCAGTAA
P17	xtrH ORF pRS426 F	GGTTTTCCCAGTCACGACGTGGGAAGAAGCACTTGATG
P18	xtrH ORF_Spacer GFP R	GTTCTTCTCCTTTACTCATTCCCCGTGTTCCGACCTCTTCCTT ATGACTCG
P19	xtrF spacer GFP pRH195 F	GTTCTTCTCCTTTACTCATTCCCCGTGTTCC
P20	xtrF pRH195 R	GTTTTTTTAATTTTAATCAAAATG
P21	xtrG spacer GFP pRH195 F	GTTCTTCTCCTTTACTCATTCCCCGTGTTCCCTCGGAATTCAA AGTCCTTC
P22	xtrG pRH195 R	GTTTTTTTAATTTTAATCAAAATGGGCAAAGATACATTCAC
P23	xtrH spacer GFP pRH195 F	GTTCTTCTCCTTTACTCATTCCCCGTGTTCCGACCTCTTCCTT ATGACTCG
P24	xtrH pRH195 R	GTTTTTTTAATTTTAATCAAAATGTATCGTATCAACAACATC
P25	GFP pRH195 F	GAATTAATAAAAGTGTTCGCTTAACGCCAAGCTTGCATGC
P26	GFP pRH195 R	GGAACACGGGGAATGAGTAAAGGAGAAGAACTTTTCACTGG
P27	cltB 5F pRS426	GGTTTTCCCAGTCACGACGCTCACCCTCGTGCTCAAC

P28	cltB 5R pyro	GACCCAACAACCATGATACCACGTGGTCAGATGTGGATAG
P29	cltB 3F pyro	CTGTCGATCATGTGGATGCTGTTCCTTGAGCAAAGAACTTG
P30	cltB 3R pRS426	CTCTCTGGAAACAGCCTTGATACTGGTGCTTTGATC
P31	cltB ORF_Spacer GFP R	GATCTCAGCGGATGAGGCTGGAACACGGGGAATGAGTAAAG GAGAAGAAC
P32	cltB 3F pyrG	GCATTGTTTGAGGCGAATTCGTTCCTTGAGCAAAGAACTTG
P33	cltB ORF_Spacer GFP pRH195 F	AGTTCTTCTCCTTTACTCATTCCCCGTGTTCCAGCCTCATCCG CTGAGATC
P34	cltB pRH195 R	GTTTTTTTAATTTTAATCAAAATGGGTGAGATCAACGAAGAG
P35	pyrG F	ATTCTGTCTGAGAGGAGGCACTGATGCG
P36	pyrG R	GAATTCGCCTCAAACAATGCTCTTCACC
P37	Pyro F	TGGTATCATGGTTGTTGGGTC
P38	Pyro R	AGCATCCACATGATCGACAG
P39	Spacer GFP F	GGAACACGGGGAATGAGTAAAGGAGAAGAACTTTTCACTGG
P40	GFP R	CTCAGACAGAATACGCCAAGCTTG
P41	pyrG tag GFP- F	GCATGCAAGCTTGGCGTATTCTGTCTGAGAGGAGGC
P42	hxtB 5F pRS426	GTAACGCCAGGGTTTTCCCAGTCACGACGCCGTCTTAGCCT GTCTTAGT
P43	hxtB ORF_Spacer GFP R	AGTTCTTCTCCTTTACTCATTCCCCGTGTTCCCGCCTTCTTAG CAATGTCC
P44	hxtB 3F pyrG	GAGCATTGTTTGAGGCGAATTCTACAGGCTTCATGTCGTTGG
P45	hxtB 3R pRS426	GCGGATAACAATTTCACACAGGAAACAGCGGTAGGATGAGTA GGCAAGA
P46	hxtB ORF_Spacer GFP pRH195 F	AGTTCTTCTCCTTTACTCATTCCCCGTGTTCCATCCTTCTCAG TCATACCCA
P47	hxtB pRH195 R	GTTTTTTTAATTTTAATCAAAATGGCGGACGGTGTCG
P48	hxtE 5F pRS426	GTTTTCCCAGTCACGACGGGAAGTGAGACGCTTTTTGGACG
P49	hxtE ORF_Spacer GFP R	CCAGAATGGCCTGCGAAAAAGGGGAACACGGGGAATGAGTA AAGGAGAAGAACTTTTCACTGG
P50	hxtE 3F pyrG	GGTGAAGAGCATTGTTTGAGGCGAATTCATCTGCCCACGTCA GCCTTCTCTC
P51	hxtE 3R pRS426	CAATTTCTCTCTGGAAACAGCCCTTTTTCGCAGGCCATTCTG G
P52	cltA ORF_ stop_pRH195_F	GAATTAATAAAAGTGTTCGCTTACTCGGAATTCAAAGTCCTTC
P53	cltB ORF_ stop_pRH195_F	GAATTAATAAAAGTGTTCGCTTAAGCCTCATCCGCTGAGATC
P54	xtrG SYBR F	CTGCTTCATCTCAACCACCA
P55	xtrG SYBR R	AGACCAGAAGACCGCTGAAA
P56	xtrH SYBR F	CTCCCAGAACTGAGCCCTAC

P57	xtrH SYBR R	ATATCCAGGTTCCCTCGTCC
P58	cltB SYBR F	GCAGTCTGTATATTCGTCTCC
P59	cltB SYBR R	GTGACGACAGAGACCTTCTG
P60	hxtB SYBR F	AACCTGAGCGGCTATGTGAT
P61	hxtB SYBR R	AACAGTGGTGGAGGCAGTCT
P62	hxtC SYBR F	CTAGCCAGTCCTCTGCCATC
P63	hxtC SYBR R	ATAAGACCAACCCCCAATCC
P64	hxtD SYBR F	GGTGCTATCGTATCGGGCTA
P65	hxtD SYBR R	TCTGGAAGGACGAACAGACC
P66	hxtE SYBR F	GGGCTCCAGAGACATTATCG
P67	hxtE SYBR R	GCCTGTCAATGCCAACTAGC
P68	tubC SYBR F	AGCTGGCGGTAACAAATACG
P69	tubC SYBR R	ACCTGATCCACCAATTCTGC
P70	cltB 1500UP F	CTCCGTCCTGATTTAATTGAGG
