# Supplementary material for: Identification and characterization of putative xylose and cellobiose transporters in Aspergillus nidulans
Source: Biotechnol Biofuels. 2016 Sep 26;9:204. doi: 10.1186/s13068-016-0611-1 (PMC5037631; doi:10.1186/s13068-016-0611-1)
Supplement: Supplementary file 5 — 10.1186/s13068-016-0611-1 Genomic DNA from the A. nidulans wild-type, ΔxtrG (AN8347), ΔxtrH (AN9173), ΔcltB (AN2814) and the double ΔcltA ΔcltB strains was extracted and digested with different restriction enzymes to confirm the deletion strains. Diagram (A.) and Southern blot (B.) of the wild-type and ΔxtrG strains when digested with SacI. A 1-kb DNA fragment from the xtrG 3′UTR (untranslated) region was used as a hybridization probe. The probe recognizes a single 10.0-kb band in the wild-type strain and a single 6.4-kb band in the ΔxtrG strain. Diagram (C.) and Southern blot (D.) of the wild-type and ΔxtrH strains when digested with EcoRI. A 1-kb DNA fragment from the xtrH 5′UTR (untranslated) region was used as a hybridization probe. The probe recognizes a single 3.4-kb band in the wild-type strain and a single 3.0-kb band in the ΔxtrH strain. Diagram (E.) and Southern blot (F.) of the wild-type and ΔcltB strains when digested with XbaI. A 1-kb DNA fragment from the cltB 5′UTR (untranslated) region was used as a hybridization probe. The probe recognizes a single 2.0-kb band in the wild-type strain and a single 3.3-kb band in the ΔcltB strain. Diagram (G.) and Southern blot (H.) of the wild-type and ΔcltA ΔcltB strains when digested with KpnI. A 1-kb DNA fragment from the cltB 3′UTR (untranslated) region was used as a hybridization probe. The probe recognizes a single 2.0-kb band in the wild-type strain and a single 2.5-kb band in the ΔxtrG strain. [file 13068_2016_611_MOESM5_ESM.pdf]

A.

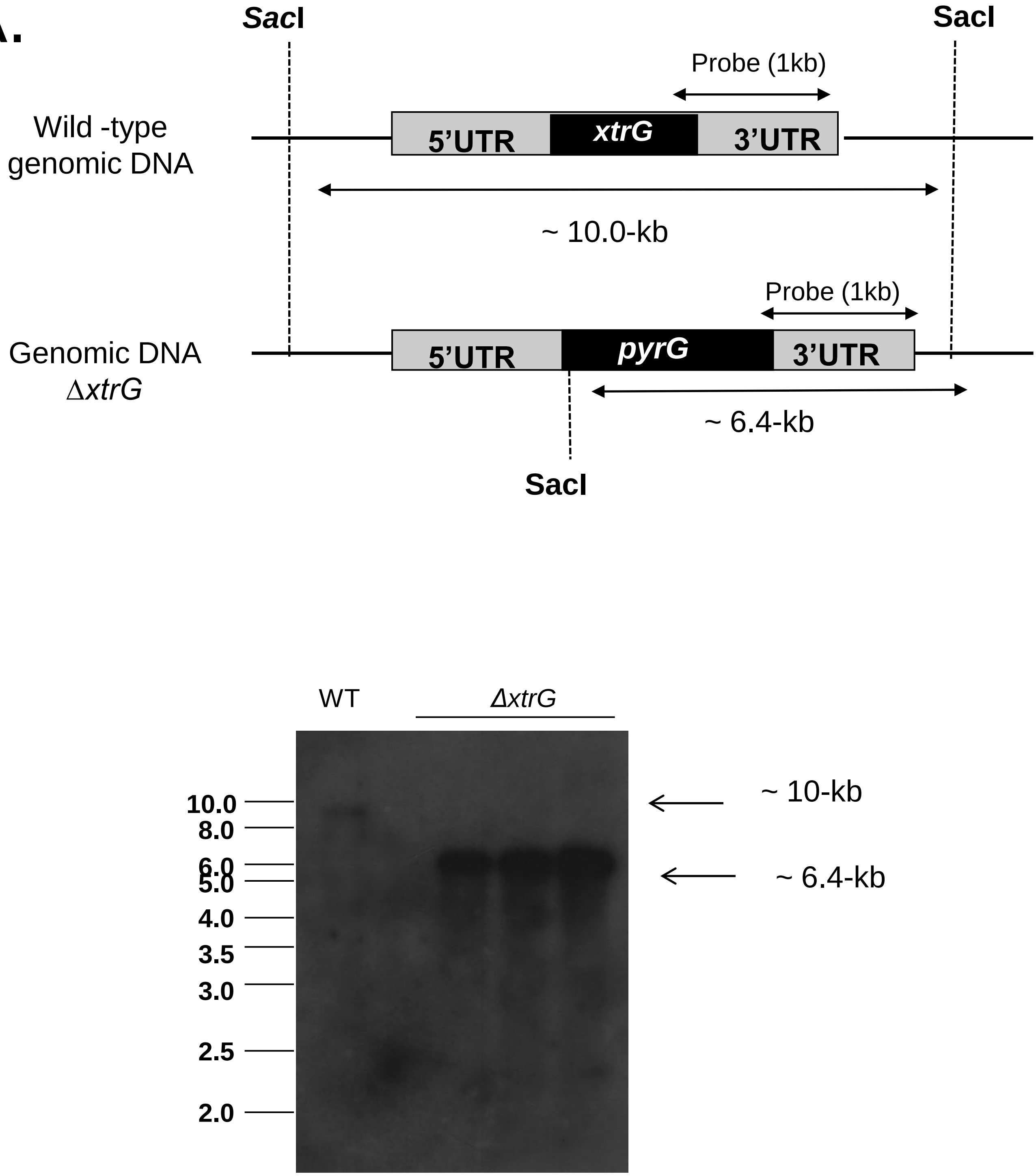

SacI
SacI
Probe (1kb)
Wild -type genomic DNA
5'UTR
xtrG
3'UTR
~ 10.0-kb
Probe (1kb)
Genomic DNA ΔxtrG
5'UTR
pyrG
3'UTR
~ 6.4-kb
SacI
WT
ΔxtrG
10.0
8.0
6.0
5.0
4.0
3.5
3.0
2.5
2.0
~ 10-kb
~ 6.4-kb

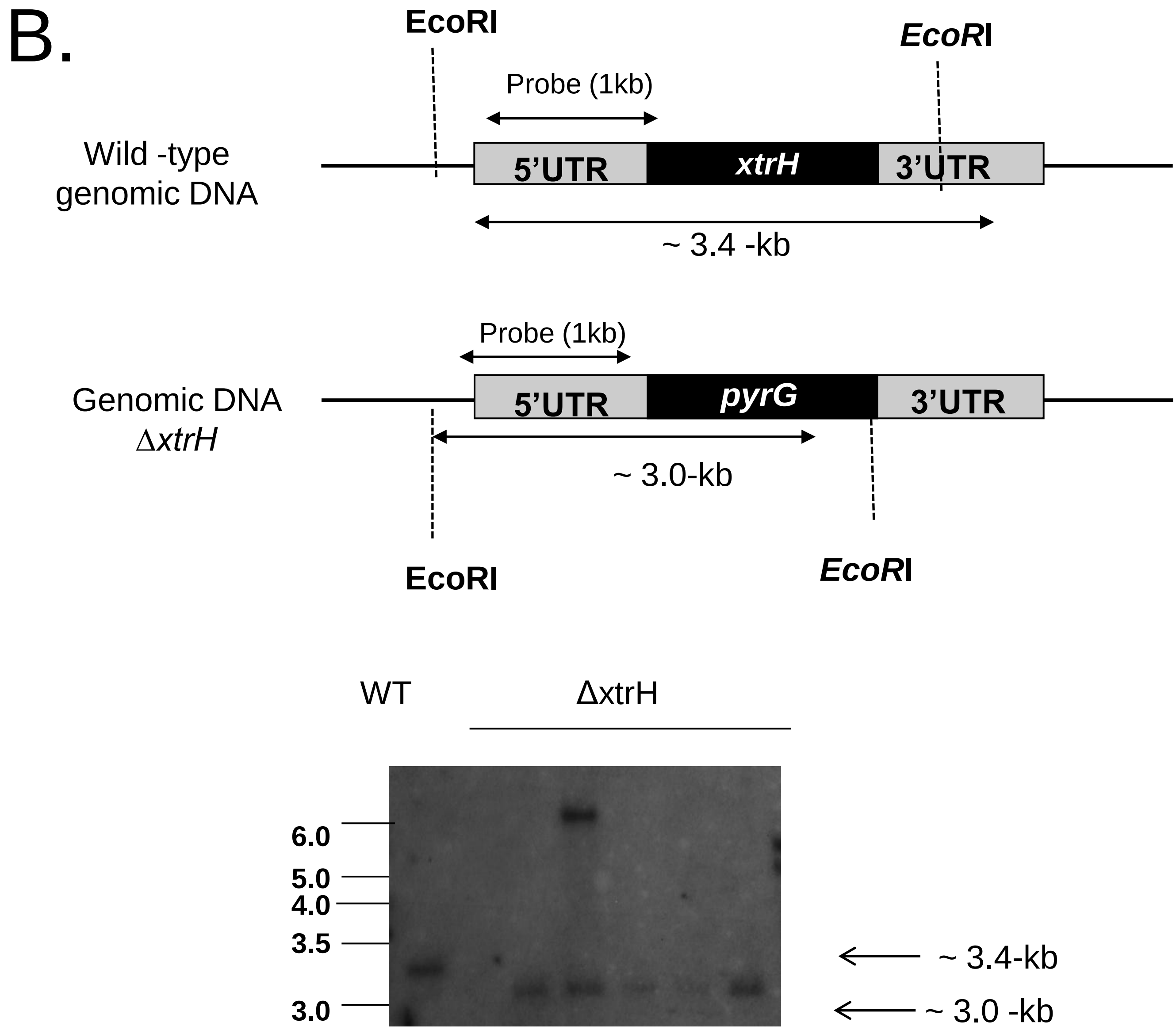
B.
EcoRI
EcoRI
Probe (1kb)
Wild -type
genomic DNA
5'UTR
xtrH
3'UTR
~ 3.4 -kb
Probe (1kb)
Genomic DNA
ΔxtrH
5'UTR
pyrG
3'UTR
~ 3.0-kb
EcoRI
EcoRI
WT
ΔxtrH
6.0
5.0
4.0
3.5
3.0
~ 3.4-kb
~ 3.0 -kb

C

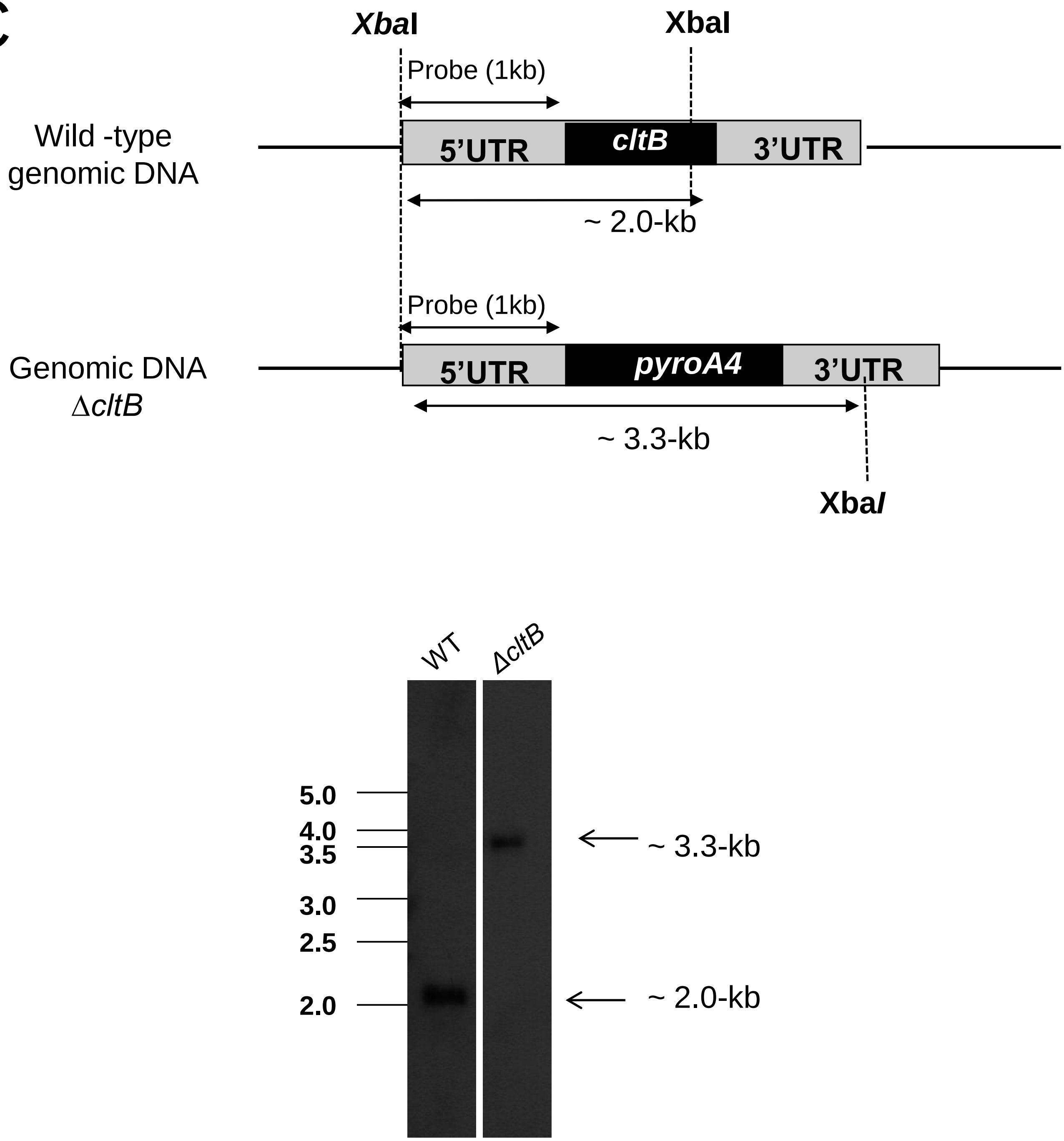
XbaI
XbaI
Probe (1kb)
Wild -type genomic DNA
5'UTR
cltB
3'UTR
~ 2.0-kb
Probe (1kb)
Genomic DNA ΔcltB
5'UTR
pyroA4
3'UTR
~ 3.3-kb
XbaI
WT
ΔcltB
5.0
4.0
3.5
3.0
2.5
2.0
~ 3.3-kb
~ 2.0-kb

D.

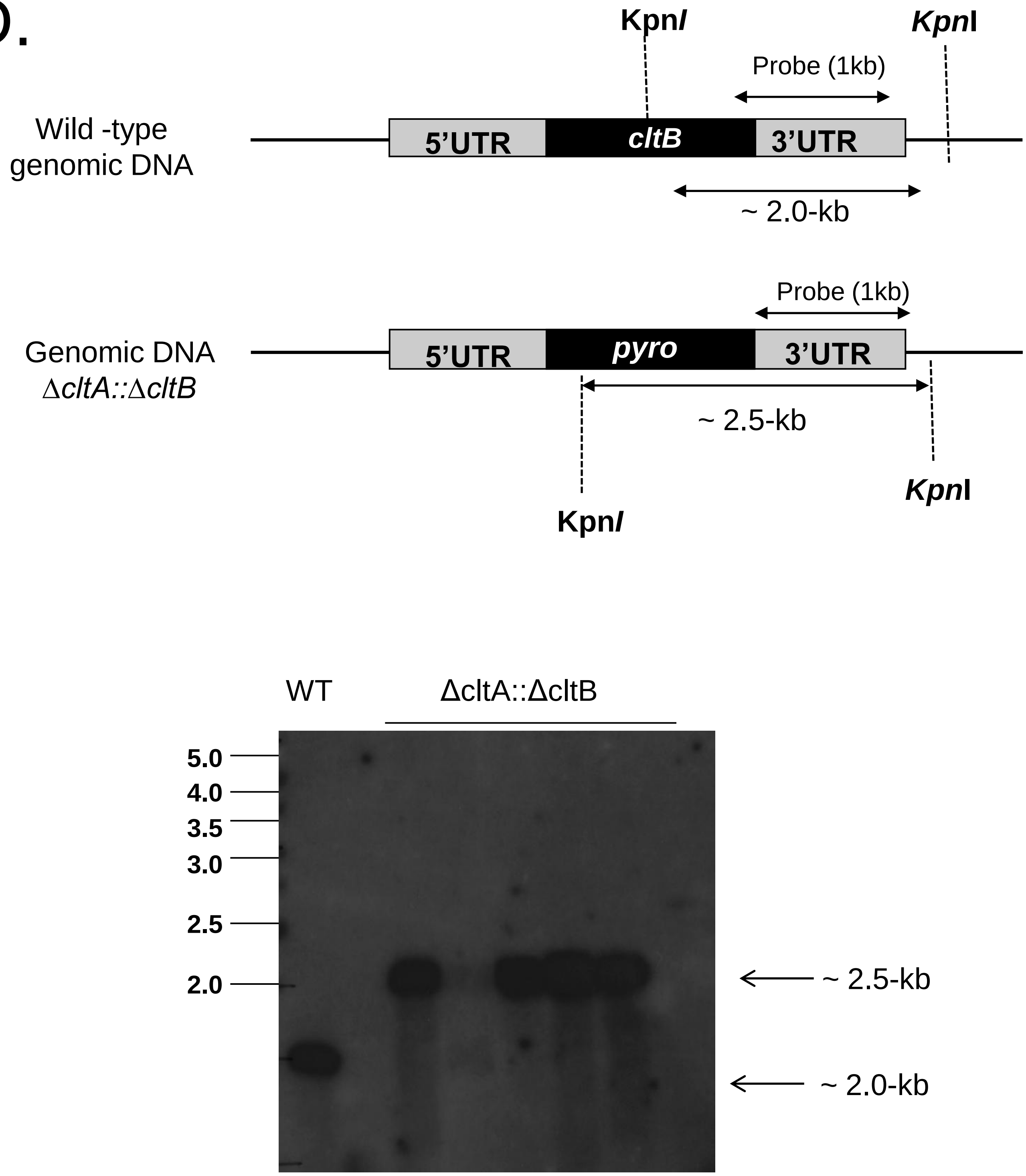
KpnI
KpnI
Probe (1kb)
Wild -type genomic DNA
5'UTR
cltB
3'UTR
~ 2.0-kb
Probe (1kb)
Genomic DNA ΔcltA::ΔcltB
5'UTR
pyro
3'UTR
~ 2.5-kb
KpnI
KpnI
WT
ΔcltA::ΔcltB
5.0
4.0
3.5
3.0
2.5
2.0
~ 2.5-kb
~ 2.0-kb

Additional File 3: Genomic DNA from the *A. nidulans* wild type, Δ*xtrG* (AN8347), Δ*xtrH* (AN9173), Δ*cltB* (AN2814) and the double Δ*cltA*::Δ*cltB* strains was extracted and digested with different restriction enzymes in order to confirm the deletion strains. Diagram and Southern blot (**A.**) of the wild-type and *ΔxtrG* strains when digested with *Sac*I. A 1-kb DNA fragment from the *xtrG* 3'UTR (untranslated) region was used as a hybridization probe. The probe recognizes a single 10.0 kb band in the wild type strain and a single 6.4 kb band in the Δ*xtrG* strain. Diagram and Southern blot (**B.**) of the wild-type and ΔxtrH strains when digested with *EcoR*I. A 1-kb DNA fragment from the *xtrH* 5'UTR (untranslated) region was used as a hybridization probe. The probe recognizes a single 3.4 kb band in the wild type strain and a single 3.0 kb band in the Δ*xtrH* strain. Diagram and Southern blot (**C.**) of the wild-type and ΔcltB strains when digested with *Xba*I. A 1-kb DNA fragment from the *cltB* 5'UTR (untranslated) region was used as a hybridization probe. The probe recognizes a single 2.0 kb band in the wild type strain and a single 3.3 kb band in the Δ*cltB* strain. Diagram and Southern blot (**D.**) of the wild-type and Δ*cltA* Δ*cltB* strains when digested with *Kpn*I. A 1-kb DNA fragment from the *cltB* 3'UTR (untranslated) region was used as a hybridization probe. The probe recognizes a single 2.0 kb band in the wild type strain and a single 2.5 kb band in the Δ*xtrG* strain.
