# Supplementary material for: Identification and characterization of putative xylose and cellobiose transporters in Aspergillus nidulans
Source: Biotechnol Biofuels. 2016 Sep 26;9:204. doi: 10.1186/s13068-016-0611-1 (PMC5037631; doi:10.1186/s13068-016-0611-1)
Supplement: Supplementary file 6 — 10.1186/s13068-016-0611-1 PCR confirmation of the ∆cltB::cltB + strain. Genomic DNA from A. nidulans wild-type, complementing strains ∆cltB::cltB + (candidates 1, 2, and 3) and deletion strain ∆cltB were isolated and used as template for PCR reactions. (A) Specific primers P70 and P31 were used to amplify a DNA fragment of about 3.1 kb. (B) Specific primers P70 and P37 were used to amplify a DNA fragment of about 3.0 kb. Lanes 1 and 7: C- negative control with no DNA as a template; lanes 2 and 8: genomic DNA from the ∆cltB::cltB candidate 1; lanes 3 and 9: genomic DNA from the ∆cltB::cltB candidate 2; lanes 4 and 10: genomic DNA from the ∆cltB::cltB candidate 3; lanes 5 and 11: genomic DNA from the ∆cltB deletion strain; lanes 6 and 12: genomic DNA from the wild-type strain. The * indicates the candidate containing the homologous integration of the complementing cassette and (C−) the negative control. [file 13068_2016_611_MOESM6_ESM.pdf]

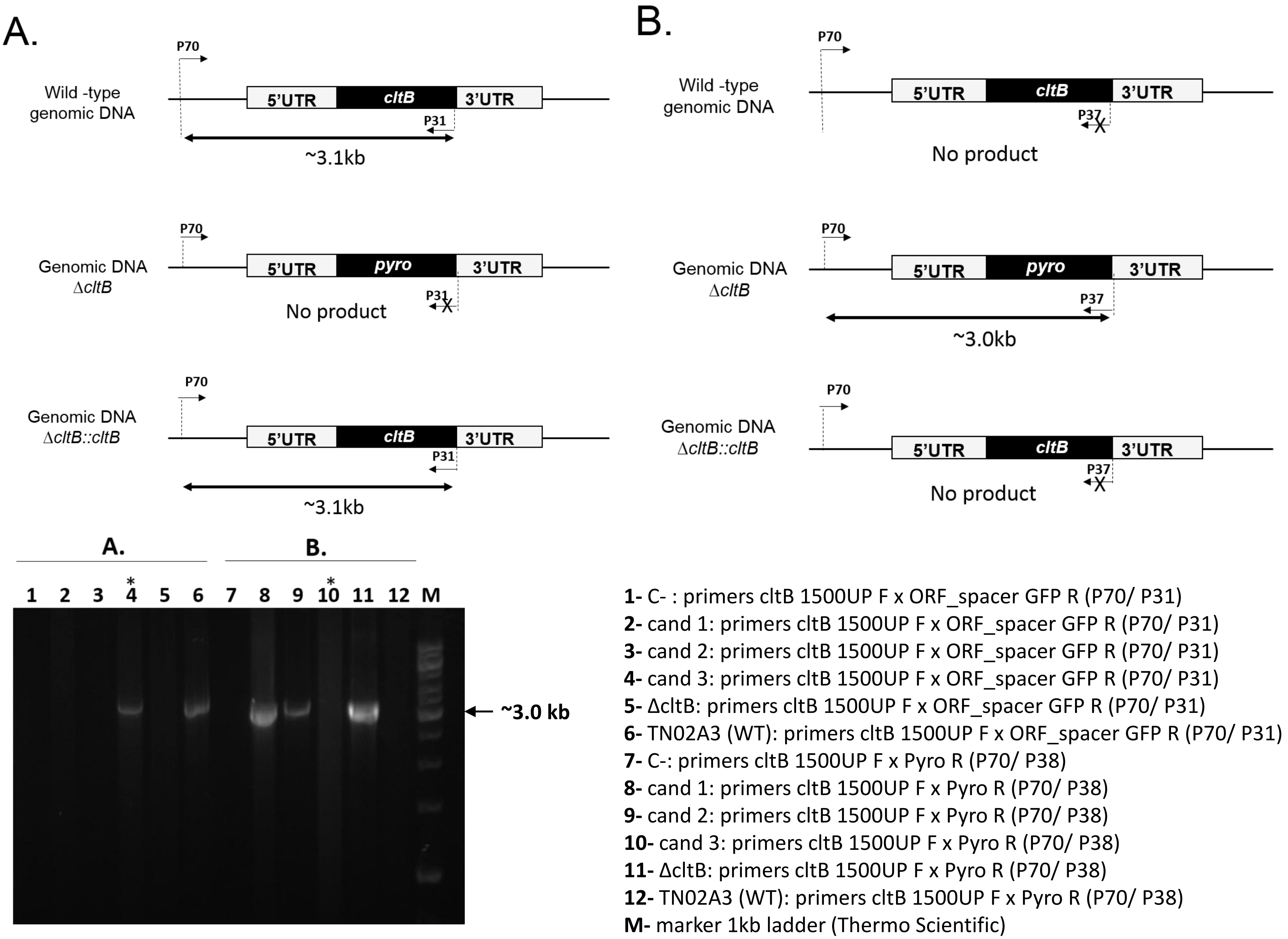

Additional file 6. PCR confirmation of the Δ*cltB::cltB*⁺ strain. Genomic DNA from *A. nidulans* wild type, complementing strains *ΔcltB::cltB*⁺ (candidates 1, 2 and 3) and deletion strain *ΔcltB* were isolated and used as template for PCR reactions. (A) Specific primers P70 and P31 were used in order to amplify a DNA fragment of about 3.1-kb. (B) Specific primers P70 and P37 were used in order to amplify a DNA fragment of about 3.0-kb. Lanes 1 and 7: C- negative control with no DNA as a template; lanes 2 and 8: genomic DNA from the *ΔcltB::cltB* candidate 1; lanes 3 and 9: genomic DNA from the *ΔcltB::cltB* candidate 2; lanes 4 and 10: genomic DNA from the *ΔcltB::cltB* candidate 3; lanes 5 and 11: genomic DNA from the *ΔcltB* deletion strain; lanes 6 and 12: genomic DNA from the wild-type strain. The * indicates the candidate containing the homologous integration of the complementing cassete and (C^-) the negative control.
